# How does integrated knowledge translation (IKT) compare to other collaborative research approaches to generating and translating knowledge? Learning from experts in the field

**DOI:** 10.1186/s12961-020-0539-6

**Published:** 2020-03-30

**Authors:** Tram Nguyen, Ian D. Graham, Kelly J. Mrklas, Sarah Bowen, Margaret Cargo, Carole A. Estabrooks, Anita Kothari, John Lavis, Ann C. Macaulay, Martha MacLeod, David Phipps, Vivian R. Ramsden, Mary J. Renfrew, Jon Salsberg, Nina Wallerstein

**Affiliations:** 1 School of Epidemiology and Public Health, Faculty of Medicine, University of Ottawa, Ottawa, Canada; 2 CanChild Centre for Childhood Disability Research, Faculty of Health Sciences, McMaster University, Hamilton, Canada; 3 Clinical Epidemiology Program, Ottawa Hospital Research Institute, Ottawa, Canada; 4 Strategic Clinical Networks™, System Innovation and Programs, Alberta Health Services, Calgary, Canada; 5 Department of Community Health Sciences, Cumming School of Medicine, University of Calgary, Calgary, Canada; 6 Applied Research and Evaluation Consultant, Nova Scotia, Canada; 7 Centre for Research and Action in Public Health, Health Research Institute, University of Canberra, Canberra, Australia; 8 Canada Research Chair, Faculty of Nursing, University of Alberta, Edmonton, Canada; 9 Faculty of Health Sciences, School of Health Studies, Western University, London, Canada; 10 Canada Research Chair in Evidence-Informed Health Systems, McMaster Health Forum, Centre for Health Economics and Policy Analysis, Department of Health Evidence, and Impact, McMaster University, Hamilton, Canada; 11 Participatory Research at McGill, Department of Family Medicine, McGill University, Montreal, Canada; 12 School of Nursing, University of Northern British Columbia, Prince George, Canada; 13 Research and Innovation Services, York University, Toronto, Canada; 14 Department of Academic Family Medicine, College of Medicine, University of Saskatchewan, Saskatoon, Canada; 15 Mother and Infant Research Unit, School of Nursing and Health Sciences, University of Dundee, Dundee, United Kingdom; 16 Graduate Entry Medical School and Health Research Institute, University of Limerick, Limerick, Ireland; 17 Center for Participatory Research, College of Population Health, University of New Mexico, Albuquerque, USA

**Keywords:** Integrated knowledge translation, Engaged scholarship, Mode 2 research, Co-production, Participatory research, Collaborative research, Partnership, Implementation science

## Abstract

**Background:**

Research funders in Canada and abroad have made substantial investments in supporting collaborative research approaches to generating and translating knowledge as it is believed to increase knowledge use. Canadian health research funders have advocated for the use of integrated knowledge translation (IKT) in health research, however, there is limited research around how IKT compares to other collaborative research approaches. Our objective was to better understand how IKT compares with engaged scholarship, Mode 2 research, co-production and participatory research by identifying the differences and similarities among them in order to provide conceptual clarity and reduce researcher and knowledge user confusion about these common approaches.

**Methods:**

We employed a qualitative descriptive method using interview data to better understand experts’ perspectives and experiences on collaborative research approaches. Participants’ responses were analysed through thematic analysis to elicit core themes. The analysis was centred around the concept of IKT, as it is the most recent approach; IKT was then compared and contrasted with engaged scholarship, Mode 2 research, co-production and participatory research. As this was an iterative process, data triangulation and member-checking were conducted with participants to ensure accuracy of the emergent themes and analysis process.

**Results:**

Differences were noted in the orientation (i.e. original purpose), historical roots (i.e. disciplinary origin) and partnership/engagement (i.e. role of partners etc.). Similarities among the approaches included (1) true partnerships rather than simple engagement, (2) focus on essential components and processes rather than labels, (3) collaborative research orientations rather than research methods, (4) core values and principles, and (5) extensive time and financial investment. Core values and principles among the approaches included co-creation, reciprocity, trust, fostering relationships, respect, co-learning, active participation, and shared decision-making in the generation and application of knowledge. All approaches require extensive time and financial investment to develop and maintain true partnerships.

**Conclusions:**

This qualitative study is the first to systematically synthesise experts’ perspectives and experiences in a comparison of collaborative research approaches. This work contributes to developing a shared understanding of collaborative research approaches to facilitate conceptual clarity in use, reporting, indexing and communication among researchers, trainees, knowledge users and stakeholders to advance IKT and implementation science.

## Introduction

Collaborative research with knowledge users is believed to be one of the best ways to support the rapid application of research evidence and generate greater impact on practice, policy, health systems and societal outcomes (i.e. effective knowledge translation (KT) and implementation practices) [1, 2]. Scholars use multiple terms, concepts, traditions, models and frameworks when describing collaborative research and practice [3, 4]. Table 1 provides a list of terms commonly employed in Canada and recognised by many funders, governments and research teams to facilitate shared understanding. Over the past two decades in Canada, health research funding agencies have promoted the funding of research partnerships as a means to promote greater uptake of research findings and thereby produce the desired research impact. This logic relates to bridging the ‘know-do’ gap [28] by focusing research on the needs of those who would use it in the real world (i.e. knowledge users) to increase knowledge use and impact. The logic also implies that, through working together on the research, researchers and knowledge users learn from each other, breaking down some of the barriers that typically divide the two communities [29, 30].

**Table 1:** Terms and definitions

Co-creation	“Co-creation - collaborative knowledge generation by academics working alongside other stakeholder - reflects a “Mode 2″ relationship (knowledge production rather than knowledge translation) between universities and society. Co-creation is widely believed to increase research impact … Co-creation emerged independently in several fields, including business studies (“value co-creation”), design science (“experience-based co-design”), computer science (“technology co-design”), and community development (“participatory research”). Key success principles included (1) a systems perspective (assuming emergence, local adaptation, and nonlinearity); (2) the framing of research as a creative enterprise with human experience at its core; and (3) an emphasis on process (the framing of the program, the nature of relationships, and governance and facilitation arrangements, especially the style of leadership and how conflict is managed).” [5] “…refers to the active involvement of end-users in various stages of the production process … However, the main difference in the definitions between co-creation and co-production is that, in line with the work of Vargo and Lusch (2004, the cocreation literature puts more emphasis on co-creation as value” [6, 7]
Co-production	“…process through which inputs used to produce a good or service are contributed by individuals who are not ‘in’ the same organization” [8] “…co-production means delivering public services in an equal and reciprocal relationship between professionals, people using services, their families and their neighbours. Where activities are co-produced in this way, both services and neighbourhoods become far more effective agents of change.” [9]
Decision-makers	“Decision-makers in the health services field can range from frontline health providers to administrators to ministers of health.” [10] “An individual who makes decisions about, or influences, health policies or practices. Decision makers can be practitioners, educators, health care administrators, elected officials (Exception: Federal elected officials), and individuals within the media, health charities, patient user groups or the private sector. They can work at the local community, municipal, provincial or national level. Decision makers are those individuals who are likely to be able to make use of the results of the research.” [11]
Dissemination	“Dissemination goes well beyond simply making research available through the traditional vehicles of journal publication and academic conference presentations. It involves a process of extracting the main messages or key implications derived from research results and communicating them to targeted groups of decision-makers and other stakeholders in a way that encourages them to factor the research implications into their work. Face-to-face communication is encouraged whenever possible.” [10] “Dissemination involves identifying the appropriate audience for research findings, and tailoring the research message and the medium to the audience to ensure optimal awareness and understanding of the message.” [12]
Engaged Scholarship	“connecting the rich resources of the university to our most pressing social, civic and ethical problems.” [13] “…collaborative form of inquiry in which academics and practitioners leverage their different perspectives and competencies to coproduce knowledge about a complex problem or phenomenon that exists under conditions of uncertainty found in the world … Our argument for engaged scholarship is based on the concept of arbitrage-a strategy of exploiting differences in the kinds of knowledge that scholars and practitioners can contribute on a problem of interest.” [14]
Implementation Science	“…the scientific study of methods to promote the systematic uptake of research findings and other evidence-based practices into routine practice, and, hence, to improve the quality and effectiveness of health services and care.” [15]
Integrated Knowledge Translation	“…represents a different way of doing research and involves active collaboration between researchers and research users in all parts of the research process, including the shaping of the research questions, decisions about the methods involvement in the data collection and tools development, interpretation of the findings and dissemination and implementation of the research results” [16] “…a way of approaching research to increase the chances that the results will be applicable to the population under study. It is a paradigm shift that focuses on engagement with end users and the context in which they work. Essentially it is a collaborative way of conducting research that involves researchers and knowledge-users, sometimes from multiple communities (e.g. clinicians, managers, policy makers, patients) working together as partners in the research process.” [17] “…a model of collaborative research, where researchers work with knowledge users who identify a problem and have the authority to implement the research recommendations” [18] “In integrated KT, stakeholders or potential research knowledge users are engaged in the entire research process. By doing integrated KT, researchers and research users work together to shape the research process by collaborating to determine the research questions, deciding on the methodology, being involved in data collection and tools development, interpreting the findings, and helping disseminate the research results. This approach, also known by such terms as collaborative research, action-oriented research, and co-production of knowledge, should produce research findings that are more likely be relevant to and used by the end users.” [19]
Knowledge Exchange	“Knowledge exchange is collaborative problem-solving between researchers and decision-makers that happens through linkage and exchange. Effective knowledge exchange involves interaction between decision-makers and researchers and results in mutual learning through the process of planning, producing, disseminating, and applying existing or new research in decision-making.” [10] “The exchange of knowledge refers to the interaction between the knowledge user and the researcher, resulting in mutual learning.” [11] “…knowledge exchange refers to the interaction between knowledge-users (those who can inform their decision-making with research) and researchers (the knowledge producers) that result in mutual learning and knowledge use.” [12]
Knowledge Translation	“…a dynamic and iterative process that includes synthesis, dissemination, exchange and ethically sound application of knowledge to improve the health of Canadians, provide more effective health services and products and strengthen the health care system.” [19] “This process takes place within a complex system of interactions between researchers and knowledge users that may vary in intensity, complexity and level of engagement depending on the nature of the research and the findings as well as the needs of the particular knowledge user.” [20]
Knowledge to Action Process	“The Knowledge to Action Process conceptualizes the relationship between knowledge creation and action, with each concept comprised of ideal phases or categories. A knowledge creation “funnel” conveys the idea that knowledge needs to be increasingly distilled before it is ready for application. The action part of the process can be thought of as a cycle leading to implementation or application of knowledge. In contrast to the knowledge funnel, the action cycle represents the activities that may be needed for knowledge application.” [19] “… IKT is about an exchange of knowledge between relevant stakeholders that results in action. To achieve this, appropriate relationships must be cultivated. The first step in this process is to identify the relevant stakeholders and to establish a common understanding of KTA [Knowledge to Action]. It is our hope that this discussion and clarification of terms, along with our presentation of a conceptual map for the KTA process, will help knowledge producers and users understand the nature of the terrain so that they can find their way through the complex, iterative, and organic process of knowledge translation.” [21]
Knowledge Synthesis	“A synthesis is an evaluation or analysis of research evidence and expert opinion on a specific topic to aid in decision-making or help decision-makers in the development of policies. It can help place the results of a single study in context by providing the overall body of research evidence. There are many forms of synthesis, ranging from very formal systematic reviews, like those carried out by the Cochrane Collaboration, to informal literature reviews.” [10] “…means the contextualization and integration of research findings of individual research studies within the larger body of knowledge on the topic. A synthesis must be reproducible and transparent in its methods, using quantitative and/or qualitative methods. It could take the form of a systematic review, follow the methods developed by the Cochrane Collaboration, result from a consensus conference or expert panel or synthesize qualitative or quantitative results. Realist syntheses, narrative syntheses, meta-analyses, meta-syntheses and practice guidelines are all forms of synthesis.” [11]
Knowledge Use	“…process by which specific research-based knowledge (science) is implemented in practice.” [22]
Knowledge Users	“…an individual who is likely to be able to use the knowledge generated through research to make informed decisions about health policies, programs and/or practices. A knowledge-user’s level of engagement in the research process may vary in intensity and complexity depending on the nature of the research and his/her information needs. A knowledge-user can be, but is not limited to, a practitioner, policy-maker, educator, decision-maker, health care administrator, community leader, or an individual in a health charity, patient group, private sector organization, or media outlet.” [11] Individuals, groups, or organisations (including patients, healthcare providers, caregivers, communities, funders, organisations and policy-makers/decision-makers, managers, researchers, trainees, industry, media/journalists) that would be considered the beneficiaries of research or who are positioned to use the research to inform their decisions about policies, programmes, treatments, interventions and practices [17, 22]
Linkage and Exchange	“…the process of ongoing interaction, collaboration and exchange of ideas between the researcher and decision-maker communities. In a specific research collaboration, it involves working together before, during and after the research program.” [10]
Mode 2 Research	“[Research that is] socially distributed, application-oriented, trans-disciplinary and subject to multiple accountabilities” [23] “[Research that is] based on the needs of end users in the health care system and is arguably a more socially accountable form of knowledge production” [24]
Partners	Knowledge users, decision-makers, stakeholders, end-users, service-users, consumers, community members, community of interest, citizens, industry, groups, funders engaged in the research process
Participatory Research	“…systematic inquiry, with the collaboration of those affected by the issue being studied, for the purposes of education andtaking action or effecting social change” [25] “…an umbrella term for a school of approaches that share a core philosophy of inclusivity and of recognizing the value of engaging in the research process (rather than including only as subjects of the research) those who are intended to be the beneficiaries, users, and stakeholders of the research. Among the approaches included within this rubric are community-based participatory research, participatory rural appraisal, empowerment evaluation, participatory action research … and forms of action research embracing a participatory philosophy” [26]
Research Partnerships	“…individuals, groups or organizations engaged in collaborative research activity involving at least one researcher (e.g., individual affiliated with an academic institution), and any stakeholder actively engaged in any part of the research process (e.g., decision or policy maker, health care administrator or leader, community agency, charities, network, patients etc.) Such arrangements might or might not be formalized at the institutional level through a memorandum of understanding.” [27]
Stakeholders	Individuals, groups or organisations with shared interest in the research; may be in the geographic locality of the research setting or may be affected by the environmental effects of the research but may not necessarily use the generated knowledge [11]

Research funders in Canada [31], Australia [32], the United Kingdom [33], the Netherlands [34] and the United States [35] have created funding opportunities to promote and support collaborative research approaches to generating and translating knowledge. Specifically, in Canada, integrated knowledge translation (IKT) has been widely promoted and used in health research [16]. Kothari et al. define IKT as “*a model of collaborative research, where researchers work with knowledge users who identify a problem and have the authority to implement the research recommendations*” [18]. Original conceptions of IKT date back to the late 1990s with work at the former Canadian Health Service Research Foundation (CHSRF) under the leadership of Jonathan Lomas, who developed the first Canadian IKT funding opportunities (then referred to as Knowledge Exchange and Linkage and Exchange funding programmes) that required health system decision-makers to be co-principle applicants on grants submitted to the CHSRF [36]; this was followed by the Canadian Institutes for Health Research (CIHR) referring to it as IKT in 2007 [16]. The CHSRF and the CIHR recognised and promoted the novelty of IKT compared to other existing collaborative research approaches through the engagement of decision-makers/policy-makers in research, who were positioned to use the outcomes to influence change, thus increasing knowledge uptake (a mandate of the CIHR). IKT and other collaborative research approaches contribute to “*the practice and science of implementation research as they provide opportunities to advance understandings of processes and factors that facilitate and hinder the development and sharing of knowledge in health systems*” [2]. However, there is a limited understanding and formal research of how IKT compares with other collaborative research approaches that have a focus on the co-creation of knowledge and its application in the real world.

To ensure the feasibility of completing this study in a timely manner, we conducted a review and preliminary synthesis of the literature [37] to identify approaches that were prevalent in the published (Medline, CINAHL, PsycINFO, Embase) and grey literatures (government reports/websites) under the following terms: (1) engaged scholarship, (2) Mode 2 research, (3) co-production and (4) participatory research. We tracked back temporally from the most recent approach (IKT, engaged scholarship, Mode 2 research, co-production, then participatory research). We selected these approaches as they are widely used globally, they are prevalent in the literature, and the research team collectively represents these comparative approaches. Definitions for these approaches are presented in Table 1. The objective of this study is to better understand how IKT compares with engaged scholarship, Mode 2 research, co-production and participatory research by identifying the differences and similarities among them. It is important to note that our goal was not about refuting or supporting IKT or any single approach but rather to bring conceptual clarity around the concepts and intents of the different approaches/traditions. Trainees, in particular, report being unclear about whether they are the same or different and, if different, in what ways. We hope to establish a shared understanding and promote a clearer description about these approaches in order to advance knowledge about the use, application, measurement and evaluation of the outcomes/impacts arising from these different varieties of ‘working together’. We recognise that there is also growing interest in ‘patient engagement’ (PE) and ‘patient and public involvement’ (PPI) in health services and, to a lesser extent, in research generation [38, 39]; however, these concepts were excluded. Our study focus was entirely on research partnerships, the last or penultimate level of engagement in most engagement frameworks, while PE and PPI typically include lesser levels of engagement such as communications/sharing, consultation/listening, deliberation, and involvement/engagement, which fall short of partnership/shared decision-making. Modes of knowledge production other than Mode 2 research were also not considered because of their less frequent use.

## Methods

### Study design

We used a qualitative descriptive method [40] with interview data to explore experts’ perspectives and experiences on the differences and similarities among IKT, engaged scholarship, Mode 2 research, co-production and participatory research. We used the COnsolidated criteria for REporting Qualitative studies (COREQ) 32-item checklist [41] to guide the reporting of this study, including the methods (data collection, data analysis) as well as the structure of results.

#### Setting

TN conducted the majority of the interviews by telephone and a few face-to-face at a convenient location for participants (home, café, workplace, office).

#### Data collection

Our primary data source were participant interviews. Interviews were semi-structured and averaged 45 min in duration (Additional file 1). The aim of the interview was to gain novel insights from participants about perceived differences and similarities among IKT, engaged scholarship, Mode 2 research, co-production and participatory research. The topics for discussion across the approaches included theoretical underpinnings (i.e. purpose and motivation) and historical development (i.e. key developers). We asked participants to identify key literature (Additional file 2) related to the various approaches, which served as our secondary data source. TN emailed the interview guide to participants 2 weeks prior to conducting the telephone/face-to-face interviews. Participants were asked to complete the interview questions independently prior to their scheduled interview. TN validated participants’ responses during the interview and took detailed notes including any additional comments. Importantly, TN utilised her learning to iterate and explore concepts of the approaches with participants, which added to the richness of the dialogue in a way that has not previously occurred. TN and IDG continued to recruit additional participants until data saturation was achieved, defined as the point at which no new themes emerged among the interviews.

### Research team and reflexivity

#### Personal characteristics

TN is a postdoctoral fellow with extensive experience in qualitative interviewing who conducted all of the interviews with interested participants. TN is supervised by IDG and has a keen interest in collaborative research, in particular IKT, as it is the focus of her postdoctoral work. Our multidisciplinary research team (*n* = 15) consisted of experienced experts (researchers (PhD) and clinicians (MD)) recognised internationally for their contributions to the field of KT and implementation science (see Table 2 for research team characteristics). The majority of our team members (*n* = 14) also contributed as study participants (*n* = 17) and authors (*n* = 14) on this paper and they may prefer their own perspective over others. However, the team collectively represented the five approaches under study, and by inviting members/experts in each realm, the research team was encouraged to take a critical stance when reviewing what was reported about each approach. We feel that, by creating a safe and collaborative exchange, experts were more likely to critically review the results and interpretations. The team views this commitment to the embedded process both as a key strength and a scientific necessity of the project and paper. In this way, we both studied and approached the work using a collaborative/IKT approach (e.g. we were ‘walking the talk’).

**Table 2:** Research team characteristics

Team members (*n* = 15)	Gender	Country	Credentials	Position
TN	F	Canada	PhD	Postdoctoral Fellow
IDG	M	Canada	PhD, FCAHS, FNYAM, FRSC	Professor
KJM	F	Canada	MSc	PhD Candidate**/**Clinician Scientist
SB	F	Canada	PhD	Professor
MC	F	Australia	PhD	Professor
CAS	F	Canada	CM, PhD, RN, FCAHS, FAAN	Professor
AK	F	Canada	PhD	Professor
JL	M	Canada	MD, PhD	Professor
ACM	F	Canada	CM, MD FCAHS FRCPC (Hon)	Professor
MM	F	Canada	PhD, RN	Professor
DP	M	Canada	PhD	Professor
VR	F	Canada	PhD, RN, BSN, MS	Professor
MJR	F	Canada	RN RM PhD FRSE	Professor
JS	M	Ireland	PhD	Professor
NW	F	United States	DrPH, MPH	Professor

Team members were aware of each other’s work and some have collaborated in the past. From the qualitative viewpoint of this study, involving diverse team members in the co-creation of the research is an optimal strategy to capture the richness of knowledge, experience and practice context, and enriches rather than detracts from the research. A primary rationale for undertaking this study was that these various approaches are typically siloed in the literature and not discussed or considered in relation to each other; this study provided an opportunity for team members to consider, analyse and describe their own approaches and view other approaches in a systematic, comparative way that had previously not been undertaken or described. This mode of dialogue offered the team a rich opportunity to see their own practice in light of others’ approaches and led to fruitful and in-depth dialogue among members and was a key design feature of the approach. Many insights were subsequently gleaned during the course of interpreting the findings and drafting the paper. Given the qualitative nature of the study, the intent was not to structure our efforts to achieve objectivity but, rather, we intended to capture and explore the depth and diversity of the approaches among those experts in conceiving/using them. The best informants for each approach were, in fact, the experts who developed them, and it is for this reason that they were recruited to participate and share their knowledge and experiences.

#### Participant selection (*n* = 17)

TN and IDG originally identified and compiled a list of 13 participants derived from the published and grey literature; an additional 10 were recommended through networking and respondent-driven sampling [42]. TN and IDG contacted participants through email (face-to-face where possible) with detailed information about the study. Contact information for participants was sought through public domains (primarily university websites, faculty directories and author contact information from key literature). Participation was voluntary and informed consent sought prior to study enrolment. Participants included renowned experts (researchers and clinicians) who have published extensively or who are recognised by their peers to have extensive expertise in the various approaches. Ultimately, a sample of 17 participants (see Table 3 for participant characteristics), primarily researchers, were enrolled in the study. We used an IKT approach to conduct the research and involved participants in every phase of the study to increase knowledge uptake. We made efforts throughout the research to create a cross-walk for participants to consider, discuss and debate the differences and similarities among approaches. All participants were offered the opportunity to interpret findings as a team and contribute as scholarly co-authors in disseminating the research.

**Table 3:** Participant characteristics

Participants (*n* = 17)	Gender	Country	Approach
1	M	Canada	IKT, CP, PR
2	M	Canada	IKT
3	F	Canada	IKT, ES, CP
4	F	Canada	Did not align with a specific approach
5	F	United Kingdom	Did not align with a specific approach
6	F	Canada	IKT
7	M	Canada	Did not align with a specific approach
8	M	Ireland	PR
9	F	Canada	PR
10	F	Canada	Did not align with a specific approach
11	F	Canada	Mode 2, IKT
12	F	Canada	CP
13	F	Canada	IKT
14	F	Australia	PR
15	F	Canada	PR
16	M	Canada	IKT
17	F	United States	PR

*CP* co-production, *ES* engaged scholarship, *IKT* integrated knowledge translation, *PR* participatory research

#### Relationship with participants

TN informed all participants of her background, interests, personal goals and reasons for conducting this study to ensure transparency. TN also kept an audit trail to ensure her bias, assumptions, personal goals, reasons and interests in conducting this study were made transparent. TN worked collaboratively with all participants on every aspect of this study, thus interested participants turned into research team members as the study evolved.

### Analysis and findings

#### Data analysis

We centred the analysis around the most recent approach, IKT, given its germinal relationship within Canadian health research funding agencies. TN and IDG conducted thematic analysis [43] of participant interviews to derive core themes from the data, which were validated by the team. An iterative process of comparing and contrasting the phrasing and meanings inherent within different perspectives was conducted. Member-checking was carried out with participants to ensure the accuracy of the emergent themes and the analysis process in an iterative manner. Our review and synthesis of the key literature within and across approaches provided additional insights and validation of the emergent themes that arose from analysis of participant interviews. No theory guided the analysis. The analysis was inductive with the following analysis categories (with related sub-categories) developed from the data by the research team: orientation (original purpose/intent, primary motivation, epistemological stance, theoretical underpinnings and theory implicit/explicit), historical roots (geographic origin, disciplinary origin, health research versus other research, and disciplinary background of early developers), and partnership/engagement (unique features, what partners are called, role of partners and power sharing). We created Table 4, which builds on the work of Bowen [44] as a comparative summary of approach characteristics. In an effort to establish face validity, we presented Table 4 at a conference workshop composed of international KT scholars [58].

**Table 4:** Summary of comparison among integrated knowledge translation, engaged scholarship, Mode 2 research, co-production and participatory research (presented here from left to right according to the timeline of their emergence from the most recent to the earliest)

Factor	Integrated knowledge translation	Engaged scholarship	Mode 2 research	Co-production	Participatory research
**Orientation**					
Scope	Research, Implementation [16]	Research/teaching scholarship [44]	Research	Research	Research
Original purpose/intent	A collaborative approach between researchers and knowledge users to increase the chances that research findings will be applicable to those under study [16]	A participative research process that expands the capabilities of scholars to gather perspectives of key stakeholders and study complex problems; the ultimate aim is to create knowledge that advances science and practice, and is more penetrating and insightful than that which is done in isolation [45]	To bring awareness to the production of knowledge within context, by increasing the flexibility to mix, coalesce and reformulate rapidly, increase the diversity of included partners, seek awareness of what the end-users see as the issues to enhance the usability and social accountability of the research, broaden the sphere of what constitutes knowledge [23]	Provides a new way of understanding and evaluating hybrid, heterogeneous arrangements that extend well beyond traditional conceptualisations of political science (policy), economics; co-production is the active involvement of consumers in various stages of the knowledge production process [46] (interchangeable with co-creation)	To address community issues in a collaborative, consultative, democratic, reflective, reflexive, dialogical and improvement-oriented fashion that builds capacity and creates actionable, ownership of findings [47]; it mobilises living knowledge of people connected together in their context and creates a common understanding of ways to act for the common good [48]
Primary motivation	Explicit focus on increasing knowledge use and impact [16]	Explicit focus on reconnecting academia with societal needs, education for democracy, civic responsibility/engagement and public scholarship [44]	Explicit focus on return on investment and increasing accountability [23, 44]	Explicit focus on increasing the effectiveness and efficiency of public services by involving consumers in the development and delivery processes	Explicit focus on social and environmental justice and a desire for impact change, particularly to benefit underserved/vulnerable citizens and communities
Epistemological stance	Neutral [44] Social constructionism [49]	Critical realist, within social construction [45, 50] (objective ontology, subjective epistemology – a process of constructing models to represent aspects of the world and comparing them with rival plausible alternates)	Described as post-modernist, post-positivist, post-industrialist [23, 51] (linkages have been drawn between Toulmin and phronesis-oriented philosophy [52]) Shares features of critical realism; counter-hegemony [5], has been viewed by others from a pragmatic perspective, as bricolage [14, 53]	Relational ontology (emphasis on interrelationships and co-constitution) or the conjoined production of one nature-culture [54]; epistemology is unstable and still evolving Some discussion of neo-materialist underpinnings	Pluralist interpretivist perspective (Aristotelian praxis, hermeneutics, constructivism, constructionism, critical theory, existentialism, pragmatism, process philosophies and phenomenology) (Northern Tradition) [55, 56] Critical pedagogy (Southern Tradition), aspects of pragmatism, pluralism, egalitarianism, Liberation theology [48] Extended epistemology of “*practical knowing*” [55] (experiential, presentational, propositional and practical ways of knowing)
Theoretical underpinnings	Initial Context for IKT: Planned Action/Change Theory [57] (set of logically interrelated concepts that systematically explain the means by which and predicts how planned change occurs in a specific environment, and helps planners control variables that increase or decrease likelihood for change); deliberate change engineering in social systems	Engaged Scholarship Diamond Model links data to theory (designed by the researcher, through engaged scholarship) [57] Model involves research design, theory building, problem formulation and problem solving within a study context, and in an iterative fashion [57] Model outlines how academics relate their teaching, discovery, integration, and application activity and retain balance between each [57]	Policy theory	Ostrom’s policy theory underlies the concept of co-production [8]	Lewin: iterative, collaborative action–reflection cycles (problem awareness, shifts in understanding, formulation of a plan of action, transformative action and progressive iterative learning, and cementing new behaviour based on effective corrective action) [48], a mode of embedded, collective self-inquiry
Theory implicit/explicit	Explicit within the KTA process, implicit as a stand-alone concept	Implicit	Implicit (fragmented, evolving)	Explicit	Explicit
**Historical roots**					
Geographic origin	Canada	United States	United Kingdom/Europe, later United States	United States, United Kingdom (post 2000)	United States United Kingdom, South America
Disciplinary origin	Health/Medicine/Nursing	Education	Philosophy	Economics, Public policy	Social sciences (Psychology in North America, Community Development, Education in South America)
Health research vs. other research	Health Research/Medicine/Nursing	General research	General research	Civil rights and social care	Civil rights and social sciences
Disciplinary background of early developers	Health Research Funders (CHSRF and CIHR), Canada Jonathan Lomas, CEO CHSRF (1997), Canada Ian Graham, VP Knowledge Translation (2007), Canada	Ernest Boyer, President of the Carnegie Foundation/Educator (1996), United States Andrew Van de Ven- Educator (2006), United States	Michael Gibbons, Physicist (1994) United Kingdom/Europe Helga Nowotny, Educator (2003) Europe	Elinor Ostrom, Economist (1978), United States Edgar Cahn, Civil rights law professor (2001), United States	Kurt Lewin, Psychologist (1946), United States/United Kingdom: ‘Northern Tradition’ Paulo Freire, Educator/Philosopher (1970), South America: ‘Southern Tradition’
**Partnership/engagement**					
Unique features	Only approach with roots in a health research and subsequently developed within health research and implementation contexts Term ‘knowledge users’ is unique to IKT (i.e. explicit focus on policy-makers/decision-makers positioned to influence change or implement the generated knowledge	Originally developed in an academic setting driven by university researchers in the United States Explicit inclusion of student partners, institutional agreements Embraces and equally emphasises all forms of scholarship (discovery, integration, application and teaching); cutting across teaching, research and service	Originally developed by educators in the United Kingdom and Europe Explicit inclusion of industry/private sector involvement as a partner; only approach to explicitly consider for-profit partnerships	Originally developed by an economist in the United States Explicit inclusion of patients (as consumers of health services), who can be considered ‘temporarily marginalised’	Originally developed in social sciences by a psychologist (Northern tradition) and an educator/philosopher (Southern Tradition) in the United States Explicit focus on social justice, power and emancipation as common outcomes Explicit focus on researcher’s humility Capacity-building is an intentional outcome
What partners are called	‘Knowledge user’, ‘Health system decision-makers’, ‘policy-makers’, ‘administrators’, ‘clinical leaders’, ‘patients’	‘Stakeholders’, ‘public members’, ‘communities’, ‘organisations’, ‘society’, ‘students’, ‘citizens’	‘End-users’, ‘industry’	‘Consumers’, ‘service users’, ‘citizens’	‘Community members’, ‘community of interest’, ‘citizens’, ‘community groups’, ‘partners’
Role of partners	‘Knowledge users’, particularly policy-makers/decision-makers and those positioned to use generated knowledge to impact change Role is negotiated (equal or equitable power and authority throughout the research process)	‘Stakeholders’ contribute diverse perspectives/’xpertise and work with researchers to resolve the conflicts that rise from them to lead to higher levels of understanding	‘End-users’ are actively engaged from the outset to ensure research agenda and objectives meet societal needs	‘Consumers’ are actively engaged as change agents (differing capabilities and interests, which sometimes may require finding synergies or trade-offs among them) in the planning and delivery of public services ‘Co-producers’ are recipients and shapers of service/goods; they may have differing capabilities and/or skills that require trade-offs/synergies	‘Community members’ (experts in lived experiences and ability to use results to influence/make local changes) and researchers (facilitators with expertise in research design/obtaining funding etc.) work together to solve a given issue
Power sharing	Equal or equitable role, power and authority throughout the research process	Leveraging expertise of stakeholders and researchers (‘arbitrage’) in co-creation of knowledge	Non-hierarchical relationship between end-users and researchers in co-reaction of knowledge	Shift in power towards service users to improve planning and delivery of public services	Empowerment and capacity-building of communities to have an equal or equitable role, power and authority throughout the research process

Adapted from Bowen 2015 [44]; Table 10.3, ‘Comparison of KT, ES, and PR’

*CHSRF* Canadian Health Services Research Foundation, *CIHR* Canadian Institutes of Health Research, *IKT* integrated knowledge translation, *KTA* Knowledge to Action

## Results

Overall, both participants and international KT scholars agreed that there is a lack of clarity among the approaches, and that differences between concepts in development, intent and context should be identified. Findings were divided into two major themes with corresponding sub-themes – (1) differences among the approaches and (2) similarities among the approaches.

### Differences among the approaches

Most participants emphasised the similarities among the approaches as the differences are minimal. However, participants identified subtle but important differences in the orientation and historical roots of the approaches as well as in partnership/engagement (see Table 4 for comparison of differences among approaches). Importantly, all participants acknowledged that the initial intent of these approaches has matured over time. Some participants suggested there may be greater variations in interpretations within, rather than between, approaches, which blurs the boundaries among the approaches. Here, we provide a brief description of the development of each approach below in order to establish context and shared understanding of the concepts. We focused on early developers of each approach as identified by participants and prevalent among the key literature.

#### IKT

IKT was first coined in 2007 by Graham [16], and subsequently advanced by the CIHR funding agency, as reflected by a participant,

“*We use this term* [IKT] *because Ian* [Graham] *so effectively popularized it in Canada.*” (P2)

However, the conceptual origins of IKT trace back to the late 1990s health policy-orientated writings of Lomas [36]. At that time, Lomas was CEO of the former Canadian Health Services Research Foundation (CHSRF), an organisation established to “*facilitate evidence-based decision making in Canada’s health sector*” [36]. His philosophy of ‘linkage and exchange’ [36, 59] set a promising course for changing the way in which research was applied to decision-making. The concept of ‘linkage and exchange’ was introduced as the Canadian government sought to systematically improve the use of sound scientific evidence by healthcare sector leaders and decision-makers/policy-makers [36] through applied research funding within health services. Through the CHSRF, conceptual awareness of linkage and exchange entered the policy realm among leaders/managers and decision-makers/policy-makers through the early 2000s. However, awareness and influence of the concept was somewhat limited, in that research addressing provider–patient or provider–client interactions lay outside the CHSRF funding mandate (the organisation did note a specific interest in building research capacity with nursing decision-makers/policy-makers) [36].

Graham and colleagues at the CIHR took up and advanced the linkage and exchange concept within the context of the uptake and use of research findings [21, 60, 61] through engagement of knowledge users (initially referred to as decision-makers) during the research process as the primary intent [62]. By the mid-2000s the CIHR had four standing funding opportunities requiring inclusion of knowledge users as co-investigators. During this time, the CIHR also refined its merit review process for these funding opportunities, which required the Review Panels to be comprised of roughly equal numbers of researchers and knowledge users. Both researchers and knowledge users assessed each proposal for (1) scientific merit and (2) potential relevance and impact. The CIHR promoted IKT as an approach rather than a study design and indicated that a collaborative approach could work with any study methodology.

While participants identified IKT as close kin to all four approaches through similar underpinnings, in the literature we reviewed, IKT is either considered epistemologically neutral or linked with a social constructivist epistemology [49]. This apparent contradiction may be explained by the focus of the relevant authors. When IKT is viewed as an approach to research as opposed to a study design, it can be considered epistemologically neutral – it can be used with any philosophy of science (meaning pragmatism, positivism, realism, etc.). When focus is on the nature of the researcher–knowledge user research partnerships, the underlying epistemological stance is social constructivism related to co-creation, mutual learning, etc., within the research partnership (as opposed to within the actual study). Building on and reformulating the linkage and exchange concept, IKT became an integral, underlying process inherent to the then-emergent Knowledge to Action Process [21, 60, 61].

IKT makes a unique contribution by adding the term ‘knowledge users’ [63], broadening inclusivity to multi-sectoral stakeholders. Formal and deliberate consideration of knowledge users and their needs forces the research team to proactively think through how change is made. This includes who (besides interested and affected parties) needs to act and who is critical to the planning of the research. IKT invites the potential and meaningful involvement of knowledge users throughout the research process, representing a significant departure from traditional research approaches. This reformulation extended well past the conceptual scope of linkage and exchange into the realm of research co-creation. Further, the conceptual evolution of IKT within the context of implementation has orientated the concept to focus on specific ‘ends’ (e.g. the improvement of health, provision of more effective health services and products, strengthening of the healthcare system, etc.).

Since the early 2010s, scholars have further explained the use of IKT and offered practical advice about how to ‘do’ IKT. IKT encourages researchers to integrate knowledge users into shaping the research and its execution/interpretation as well as the use of its products, in practice [64, 65]. With time, the articulation of IKT has also become more explicit with respect to outcomes/impact, return on investment, and benefits to society as a form of public/social accountability for the investment of public funds in health research [66–68]. For example, IKT explicitly emphasises the inclusion of decision-makers/policy-makers who are positioned to use the generated knowledge to impact change. Examples of participant responses included,

“*For me IKT must include policy makers/managers who are in positions to use the results to change policies or make decisions. Effective IKT requires inclusion of policy makers and decision makers from the beginning to help shape the question(s) and to recognize they have a very important role to play in using results to make a difference. Participatory and community-based participatory research etc. do not state this explicitly. Ian Graham with IKT shifted my insight to realize the importance of getting policy/decision makers to be involved (small or big policy people) if you want to make an impact (consciously aware for IKT).*” (P9).

“*IKT involves decision makers in many stages of the research process (generation of research). Decision makers who are partners have some kind of power to help implement the findings (chosen specially because of their position/power not because they are disempowered).*” (P13)

Interestingly, since the early 2010s, documented use of IKT has moved outside the health domain towards a conceptual guide for the conduct of research [69–73] in non-health research domains (e.g. engineering, environmental science, stem cell research, technology development, public transportation, among others).

#### Engaged scholarship

The concept of engaged scholarship was articulated by the educator Ernest Boyer in the 1990s in the United States [74]. Boyer drew attention to the growing disconnect between academics and societal needs through the promotion of public engagement in research [75, 76]. During this time, there was a major movement and urgent call for American universities to ‘return to their roots’, and to conduct research that meets societal needs rather than the needs of individuals (researchers, students, faculties within the university) or institutions [77–81]. The roots of engaged scholarship stem from a re-evaluation of what we think about scholarship (how do we engage people) and the social issues that we care about (how do we make academia more relevant to social issues). Thus, there are two levels of engaged scholarship – (1) transformation of academia and re-invigorating existing academic systems and structures and (2) encouraging researchers to reconnect research with social issues through engagement.

The initial intent of engaged scholarship was education for democracy, civic responsibility/engagement and public scholarship facilitated by university researchers [81–84]. Importantly, Boyer posits that discovery (‘pure’ or ‘basic’ research) is only one form of scholarship and offers integration, application and teaching as three other complementary forms [76, 85, 86]. A participant stated:

“*Engaged scholarship is unique as it engages all forms of scholarship - not only* ‘*discovery*’ *research, which is how research is often defined in the academic setting. It also recognizes and urges valuing of integration of research, finding applications for research (as well as teaching).*” (P10)

The embracing of all forms of scholarship (discovery, integration, application, teaching) is a unique contribution. This framing also cuts across the traditional academic evaluation criteria of teaching, research and service. Van de Ven later introduced the concept of ‘arbitrage’ in engaged scholarship as “*a strategy of exploiting differences in the kinds of knowledge that scholars and practitioners can contribute on a problem of interest*” [14]. As a way of bridging the gap between theory and practice, Van de Ven sought to leverage the expertise of scholars and practitioners to enhance knowledge use and implementation practices [45, 87].

#### Mode 2 research

Gibbons et al. [88, 89] (with further developments by Nowotny et al. [51, 90]) proposed a new form of discovery they termed Mode 2 research in the 1990s in the United Kingdom/Europe. A Mode 2 approach to discovery promotes research that is reflexive, transdisciplinary, relevant, scientifically valid, issue-driven, context-specific and socially robust through the engagement of end-users (including industry to consider for-profit partnerships) in the research process [23, 44, 81]. The initial intent of Mode 2 research was to create knowledge based on the needs of end-users and increase researcher and social accountability. It stands in stark contrast to Mode 1 research, the traditional view of ‘objective science’ that canonises the autonomy of researchers and institutions (and is characterised in a system of academic tenure, peer-reviewed publications and criteria for validity, scientific expertise) and is frequently carried out in disciplinary silos [44]. Mode 1 research is driven by a more purely discovery model [89]. More recently, scholars have introduced a Mode 3 form of discovery, in which teams work simultaneously cross Modes 1 and 2, are adaptive to current problem contexts, co-evolve different knowledge and innovations, participate in civic engagements (beyond university–business–government) and link systems and system theory [91, 92]. However, we have restricted our work to Mode 2, which clearly underpins an IKT approach and more commonly appears in the health literature as being a foundation for partnered forms of research.

#### Co-production

Co-production is a term first coined by the economist Elinor Ostrom in the late 1970s in the United States [93, 94]. The initial intent of co-production was to ensure that consumers or citizens were involved in the production as well as consumption of public services [95–99]. Explicit inclusion of patients (as consumers of health services, who can be considered ‘temporarily marginalised’) is a unique feature of co-production. Edgar Cahn, a civil rights law professor, further developed co-production to incorporate principles of social justice and equity [100]. Key contributors to the development of co-production include Jasanoff [101, 102] (science and technology) and Coot [103] (health services). Co-production combines academic insight and scientific excellence with public benefit, blurring the boundaries between ‘pure’ and ‘applied’ research, and aligning closely and building upon participatory research [76].

#### Participatory research

Participatory research predates all other approaches we are considering; it has the largest body of literature and longest lineage among the approaches. Participatory research has its origins in social action research and an emancipatory philosophy. Historically, there are two traditions of participatory research. First, there is the Northern Tradition [104, 105] or ‘action research’ developed by Lewin in the 1940s in the United Kingdom and United States. The aim of the Northern Tradition was to promote cyclical practices of involving community members and workers in collaborative efforts towards organisational and social change, rather than on, for or about them. Second, there is the Southern Tradition [106] or ‘emancipatory research’, inspired by Freire in the 1970s in South America. The aim of the Southern Tradition was to promote practices and strategies to engage oppressed, marginalised, disadvantaged or disempowered communities within a dominant society, primarily Latin America, Africa and Asia, to affect policy change. The initial intent of the Southern arms of participatory research was on social justice (generating knowledge that can be utilised for social change), power and emancipation (this also includes knowledge utilisation and self-determination) for interested or affected patients, individuals or communities. In the United States, this emancipatory approach has been most frequently adopted by community-based participatory research, or in Canada, by those involved in community-based research. Community-based participatory research has been equally invested in achieving research implementation and impact (as seen through National Institutes of Health, Centres for Disease and Control and Prevention, and Patient-Centered Outcomes Research Institute funding). Recent writers from Indigenous perspectives [107] and from the global south [108, 109] have called for knowledge democracy and cognitive justice that honours community expertise as equal to academic expertise in co-generating research knowledge, and that recognises the authority of communities to direct the research design and methodologies. Capacity-building is an outcome of all approaches but is intentional in participatory research.

Well-established in public health and community-based health initiatives, participatory research is also an emerging approach in other disciplines beyond health and extends to policy and health services research [44]. The three key motivations of participatory research include social and environmental justice and a desire for impactful change (particularly to be accountable towards communities and to benefit underserved/vulnerable citizens and communities), translating knowledge into action (knowledge utilisation towards equity and social justice), and self-determination [26]. Participants also pointed out that the humility of the researchers, including cultural humility, is an integral aspect of the participatory research process. This involves reflection on one’s positionality of power and privilege so that issues of power sharing can be directly addressed in partnering processes [110]. Reflection about one’s power and privilege was not made explicit in other approaches, although the emphasis on equal but different roles found in engaged scholarship promotes a form of humility. It is important to recognise that there are many contributors who have informed the development of participatory research that are not discussed here (Allen [111], Argyris [112], Arnstein [113], Borda [114], Cargo [26], Dewey [115–117], Duran [118], Green [119], Habermas [120], Hall [121, 122], Israel [123–125], Jagosh [126, 127], Kemmis [128], Kuhn [129, 130], LeMaster [111], Macaulay [131, 132], Mercer [133], Minkler [134–139], Ramsden [140], Salsberg [141–143], Schon [112], Selener [144], McTaggart [128], Viswanathan [145], Wallerstein [146–149], Wright [150], Westfall [111], Foote Whyte [151]).

### Similarities among the approaches

Analysis of participant responses and the key literature revealed several similarities among the approaches, for example, (1) true partnerships rather than simple engagement; (2) a focus on essential components and process rather than labels; (3) collaborative research orientations rather than research methods; (4) core values and principles, and (5) extensive time and financial investment. These are discussed below.

#### True partnerships rather than simple engagement

Most participants expressed the perspective that all approaches shared a commonality in facilitating true partnerships, meaning a deep relationship between researchers and knowledge users during the entire research process to ensure the research is beneficial for all parties involved. The knowledge and contributions of researchers and knowledge users are different but equally valued. This understanding of bi- or multi-directional learning has been strengthened by Indigenous theorists, who do not use the term knowledge users as much as co-knowledge creators and producers. In this perspective, knowledge users are not passive subjects but active participants and experts in their own right (lived experience), while researchers are experts in research design, coordinating research activities and securing funding.

“*In a true partnership (and this reflects an engaged scholarship perspective), all perspectives/expertise are considered equally valuable – but different. We do not expect knowledge users to be researchers or bring research expertise, for example. All partners are involved from the earliest stage of the research (e.g. determining priority topics and research questions) in meaningful ways. Importantly, power is shared. Partnership involves creating an environment where diverse perspectives, including differences, are discussed in depth, resulting in a higher level of understanding.*” (P10)

This quote highlights a power shift towards shared decision-making, equality and co-learning, recognising the expertise and unique contributions of all those involved in the co-creation of knowledge. Some participants noted that not all partnerships are authentic. Often, knowledge user engagement relates solely to consultation or feedback at one point in time where knowledge users do not have an active role in the decision-making process.

“*Engagement refers more to consulting, whereas partnership implies a stronger and tighter relationship.*” (P13)

However, another participant stated that engagement occurs along a spectrum or continuum ranging from passive to active participation as the term ‘engagement’ has many different meanings for different people.

#### Focus on essential components and process rather than labels

Aligning with the previous theme of true partnerships, another theme that emerged was the focus on essential components (core values and principles) and process (team interactions and dynamics) for working together in a meaningful way rather than labelling an approach. A participant summarised the essence of all the approaches as:

“*It’s all about working together to solve problems that matter to people and making a difference. It’s about making sure the benefits of the research are applied and the outcomes realized to make things better.*” (P16)

Another participant put it this way,

“[It’s] *not so much what we call it but it’s what we do.*” (P8)

While participants recognised the need for labelling an approach (particularly among trainees) in order to provide guidance for methods, scientific credibility and rigor, many participants voiced their lack of enthusiasm for labels. Participants expressed concern with the focus on labels with the changing terminology:

“*I have found, over the years, that there are all sorts of new methods or approach that purport to be different, but often are marginally so. They may be coined as something different in order for someone to be seen to create something new, or to be able to sell an approach. Often it is repackaging with a new name. People want to label things and then they promote it.*” (P4)

Many participants found it difficult to align themselves with a single approach. Thus, they focused on the collaborative process of working with knowledge users in meaningful partnership, using whatever approach (or combination of elements from various approaches) they deemed suitable to achieve the objectives of a particular study [152, 153].

“*My overall comment is that the similarities outweigh the differences. In my opinion the KEY item is the level of true partnership throughout all phases of the research*” and “[I’m] *not excited by all this academic terminology,* [I] *feel strongly that the key issue is equity of team members; it may vary from project to project. How the team decides to work … it depends on all your other factors: research question; the funding source that drives a lot of this; expertise of the team; who you’ve got on the team; it depends on length of the projects; who owns the research and who can access it; every team is different, it is not a cookie cutter approach.*” (P9)

#### Collaborative research approaches rather than research methods

All participants described all of the approaches as collaborative research orientations (plans and procedures for research that consist of philosophical underpinnings, broad assumptions/worldviews and integrity), rather than research methods (the forms of data collection, analysis and interpretation) or research methodologies (theoretical study or analysis of the methods) [154, 155]. All of the approaches challenge the traditional academic practice of purely researcher-driven research in favour of engaging knowledge users in the research process to improve research relevance, implementation practices and impact. A participant stated:

“*I use the same theory/principles – i.e. the research end users and researchers come together to develop the question, develop the protocol and complete the study, interpret results, disseminate strategies. All team members are equal partners.*” (P12)

Participants emphasised the importance of recognising distinctions between research orientations, methods and methodologies as these terms are often used interchangeably, which can cause confusion.

#### Core values and principles

Core values and principles common among the approaches include co-creation, reciprocity, trust, fostering relationships, collaboration, respect, co-learning, active participation, democratisation of knowledge and shared decision-making in the generation and application of knowledge.

“*Inclusion of knowledge users is one of the core principles or values. People want to be heard and a healthcare system that is responsive to their needs and not the needs of researchers.*” (P16)

“*Involving patients and communities; principles are the same involving patients in shared decisions making for clinical care and for research.*” (P9)

“*The goal of answering questions of mutual concern, engagement with the ideas and the work, and motivation to collectively enable change are closely linked to the co-creation/co-production model. This work model may not be ideal for all projects, but in health care it is ideal for most.*” (P3)

#### Extensive time and financial investment

All participants agreed that extensive time and financial investment must be recognised to establish and sustain true partnerships as it is often overlooked by funders.

“*Research that is co-created takes time, patience, energy and commitment. The engagement of individuals/patients, organizations and/or communities has become increasingly important in all aspects of the research process. Research that is co-created with individuals/patients, organizations and/or communities is designed to improve health and well-being and to minimize health disparities*” (P15)

“*It took a long time for me to be ready to undertake such work* [collaborative work] *due to time and resources*.” (P11)

Participants felt that extensive time, effort, commitment and resources are required in building rapport and trust with knowledge users prior to, during and beyond the completion of the study. Many participants also discussed the challenges of securing funding because of limited funding opportunities and limited rewards for undertaking collaborative work, particularly in the early stages of a researcher’s career.

## Discussion

In summary, most participants emphasised the similarities among the approaches, as the differences seemed minimal. Some participants also recognised that, while the approaches may share common principles, the way they are enacted is contextual, and is related to the epistemological stance of those involved and the particular project. Overall, the key contribution of this paper is to advance the practice and science of IKT by making concrete and explicit its differences and similarities compared to other collaborative research approaches. Identifying the similarities, with differences in origin and emphasis, among collaborative research approaches is novel and has added value in informing future research and practice as this is the first study to bring experts together to reflect on these approaches. Future research should build on this work and systematically review the advantages as well as the disadvantages of each of these approaches.

There are four key distinguishing features of IKT worth noting. First, IKT is the only approach originally developed in a health research context (Medicine and Nursing) by health research funders in Canada. Second, IKT is either considered epistemologically neutral or linked with a social constructivist epistemology [49] in the literature we reviewed. Third, IKT has typically not been about emancipation of knowledge users but rather focused on increasing knowledge use, implementation and impact. Fourth, IKT makes explicit the engagement of knowledge users throughout the research process, which forces the research team to proactively think through how change is made, and therefore who needs to be involved in the planning of the research to enact change. Further, the conceptual evolution of IKT within the context of implementation has orientated the concept to specific ‘ends’ (e.g. the improvement of health, provision of more effective health services and products, strengthening of the healthcare system, etc.).

Similarities among the approaches included true partnerships rather than simple engagement, a focus on essential components and process rather than labels, collaborative research orientations rather than fixed research methods, and core values and principles. Core values and principles common among the approaches include co-creation, reciprocity, trust, fostering relationships, collaboration, respect, co-learning, active participation, democratisation of knowledge, and shared decision-making in the generation and application of knowledge. All approaches were considered by participants to require extensive time and financial investment to develop and maintain true partnerships.

There was consensus in some areas (e.g. IKT is the only approach originally developed in a health context by health research funders in Canada) and negotiated agreement in others among the participants (e.g. IKT is either considered epistemologically neutral or linked with a social constructivist epistemology). Many participants found it challenging to align themselves with a single approach as they used a combination of principles from multiple approaches (embracing the diversity among the approaches and taking the best from whichever approach or approaches and make it work with the funding available). Instead of focusing on labelling an approach, participants emphasised the need to focus on the collaborative process (i.e. objectives, context, roles, who to engage, when to engage, power sharing, and how to initiate and continue the engagement) [152, 153]. Participants agreed that there is no recipe for collaborative research as it is a dynamic process and we need to learn through hands-on experience. Participants stated that engagement occurred along a continuum of participation [156] and researchers should provide descriptions of teams and shared decision-making processes when reporting collaborative research projects (i.e. project governance and decision-making process, team functioning, roles, contributions). We encourage researchers and trainees to use the key dimensions proposed in Table 4 as a guide for reporting.

Participants raised other important questions, including “*What constitutes true partnership or minimum engagement?*”, “*What is participation?*”, “*Is true partnership necessary for all approaches?*”, “*What do ‘core’ or ‘valuable’ principles mean in relation to the marginal differences which could be important to acknowledge? Do we ask users what is valuable? Does it depend on the type of question?*” Addressing these questions will begin to provide added clarity around the differences among the approaches. Participants expressed that, in order to promote the development of true partnerships in research, additional effort and revisions to current university standards for productivity and academic reward is needed to acknowledge the extensive time, effort and resource investment required for establishing true partnerships [157].

### Potential use of findings for researchers, trainees and other knowledge users/stakeholders

This qualitative descriptive study is the first to systematically synthesise experts’ perspectives and experiences in a comparison of various approaches to collaborative research, using an IKT lens. It is also the first to bring experts in the field together to synthesise the similarities and differences among the approaches. For researchers and trainees, this work offers novel insights and foundational knowledge as a starting point for an inter-disciplinary discussion/dialogue about the nature, characteristics, measurement and evaluation of various collaborative research approaches in a more refined way. For example, the results of this study provide novice researchers and trainees with a common working document to help familiarise themselves with the terms and concepts used in collaborative research to inform future research that will advance IKT science and practice.

Knowledge users and stakeholders may find the explicit description of principles and values of collaborative research helpful in making decisions around how and when they would like to be engaged to facilitate meaningful engagement. The themes identified in this study may provide a foundation and entry point to assist knowledge users and stakeholders in initiating discussions about what constitutes engagement, collaborative research or co-creation with researchers prior to agreeing to partner with them. For funding agencies and decision-makers/policy-makers, the description of the approaches and the similarities and differences may be of use when designing partnered funding calls.

### Limitations

It is important to note that these results are primarily based on the views of the participants and a review of the key literature. The process of synthesising the differences and similarities between IKT and other collaborative research approaches is complex and multi-layered. It was challenging to integrate all of the participants’ views and feedback to establish negotiated agreement. Readers should be mindful when interpreting the results of this study and resist oversimplification and generalisation, as exemplified by the level of detail provided in Table 4. We acknowledge that the participants are predominately Canadian researchers, but this is not surprising given our team’s focus on IKT as well as the broader Canadian funder-linked orientation to IKT. We migrated this limitation by conducting a face validity check with the international community of KT scholars who identified similar concerns as our participants regarding a lack of clarity among the approaches. We also attempted to recruit international experts, yet many were unable to enrol in the study. Those who declined participation stated scheduling conflicts and other previous commitments as the hindrance. Furthermore, for several of the historically mature approaches, source experts were deceased.

Additionally, this paper is focused on researchers’ perspectives and we would have likely gained different insights had we included community members and other knowledge users with experiences with the different approaches. Thus, we recognise that our results and discussion may be skewed in addressing only the needs of researchers and not those of partners. We also acknowledge that the selection of approaches is not inclusive of all collaborative research (i.e. PE, PPI and other modes of knowledge production beyond Mode 2). We selected approaches most prevalent in the literature as a starting point and to keep the study manageable. Nevertheless, we suggest that the comparison of IKT with the included four other dominant approaches remains a useful starting point for researchers, trainees, and other knowledge users and stakeholders. Lastly, we did not take a systematic approach to the literature review because the focus was on experts and their identification of relevant literature.

### Directions for future research

Primary data collection from community members, knowledge users and stakeholders who engage in collaborative research should also be undertaken to understand the issues and challenges from their perspectives, which may differ from those of theorists in the field. There is a need for additional comparative effectiveness research to examine outcomes and impact across [158–160] these approaches to reveal whether and how outcomes differ by approach. There is also a need for research into how current university standards for productivity and promotion influences researchers’ intent in undertaking collaborative research and how institutional disincentives to engage in collaborative research can be overcome [157].

## Conclusions

Overall, the results of this study suggest that IKT, engaged scholarship, Mode 2 research, co-production and participatory research approaches are more similar than different to each other. All facilitate true partnerships (shifting to shared decision-making that values contributions from all partners equally), require extensive time and financial investment, and share core values and principles (inclusion of knowledge users throughout the research process, co-creation of knowledge, reciprocity, shared decision-making, active participation and democratisation of knowledge production). Important differences were noted in the orientation and historical roots as well as in partnership/engagement among the approaches. This study is the first to bring experts together to share insights/experiences and reflect on what they think about multiple collaborative research approaches. This work contributes to developing a shared understanding of collaborative approaches to facilitate clarity in use, reporting, indexing and communication among researchers, trainees, knowledge users and stakeholders to advance IKT and implementation science as well as knowledge around collaborative research.

## Supplementary information

**Additional file 1.** Interview guide.

**Additional file 2.** Key literature.

## Data Availability

Not applicable to protect participants’ anonymity/confidentiality.

## Abbreviations

CHSRF: Canadian Health Services Research Foundation
CIHR: Canadian Institutes of Health Research
IKT: Integrated knowledge translation
KT: Knowledge translation
PE: Patient engagement
PPI: Patient and public involvement

## References

[1] Gagliardi AR, Berta W, Kothari A, Boyko J, Urquhart R (2015). Integrated knowledge translation (IKT) in health care: a scoping review. Implement Sci.

[2] Jull J, Giles A, Graham ID (2017). Community-based participatory research and integrated knowledge translation: advancing the co-creation of knowledge. Implement Sci.

[3] Straus SE, Tetroe J, Graham ID (2013). Knowledge translation in healthcare: moving from evidence to practice.

[4] Drahota A, Meza RD, Brikho B (2016). Community-academic partnerships: a systematic review of the state of the literature and recommendations for future research. Milbank Q.

[5] Greenhalgh T, Jackson C, Shaw S, Janamian T (2016). Achieving research impact through co-creation in community-based health services: literature review and case study. Milbank Q..

[6] Voorberg WH, Bekkers VJ, Tummers LG (2015). A systematic review of co-creation and co-production: Embarking on the social innovation journey. Public Manag Rev.

[7] Vargo SL, Lusch RF (2004). Evolving to a new dominant logic for marketing. J Mark.

[8] Ostrom E (1996). Crossing the great divide: coproduction, synergy, and development. World Dev.

[9] Boyle D, Harris M (2009). The challenge of co-production–How equal partnership between professionals and the public are crucial to improving public services.

[10] Canadian Foundation for Healthcare Improvement (formerly the Canadian Health Services Research Foundation) (2018). Glossary of knowledge exchange terms.

[11] Canadian Institutes of Health Research (CIHR) (2019). Glossary of funding-related terms.

[12] Graham ID, Tetroe J, Pearson A (2014). Turning knowledge into action: practical guidance on how to do integrated knowledge translation research.

[13] Boyer EL (1996). The scholarship of engagement. J Public Serv Outreach.

[14] Van de Ven AH, Johnson P (2006). Knowledge for theory and practice. Acad Manag Rev.

[15] Eccles MP, Mittman BS (2006). Welcome to implementation science. Implement Sci.

[16] Graham ID, Tetroe J (2007). CIHR research: how to translate health research knowledge into effective healthcare action. Healthc Q.

[17] Graham ID, Tetroe J, MacLean RK, Graham ID, Tetroe J, Pearson A (2014). Chapter 1: some basics of integrated knowledge translation research. Turning knowledge into action: practical guidance on how to do integrated knowledge translation research.

[18] Kothari A, McCutcheon C, Graham ID, for the IKT Research Network (2017). Defining integrated knowledge translation and moving forward: a response to recent commentaries. Int J Health Policy Manag.

[19] Canadian Institutes of Health Research (CIHR) (2016). Knowledge translation at CIHR.

[20] Graham ID, Tetroe J. Getting Evidence into Policy and Practice: Perspective of a Health Research Funder. J Can Acad Child Adolesc Psychiatry. 2009;18(1):46–50. PMC2651211 19270848

[21] Graham ID, Logan J, Harrison MB, Straus SE, Tetroe J, Caswell W, Robinson N (2006). Lost in knowledge translation: time for a map?. J Contin Educ Heal Prof.

[22] Estabrooks CA, Floyd JA, Scott-Findlay S, O’leary KA, Gushta M (2003). Individual determinants of research utilization: a systematic review. J Adv Nurs.

[23] Gibbons M, Limoges C, Nowotny H, Schwartzman S, Scott P, Trow M (1994). The new production of knowledge: the dynamics of science and research in contemporary societies.

[24] Estabrooks CA, Norton P, Birdsell JM, Newton MS, Adewale AJ, Thornley R (2008). Knowledge translation and research careers: Mode I and Mode II activity among health researchers. Res Policy.

[25] Green LW, George A, Daniel M, Frankish J, Herbert CJ, et al. Participatory research in health promotion. Ottawa: The Royal Society of Canada; 1995.

[26] Cargo M, Mercer SL (2008). The value and challenges of participatory research: strengthening its practice. Annu Rev Public Health.

[27] Mrklas KJ (2017). A scoping review of available tools for assessing integrated knowledge translation research or health research partnership impact.

[28] Bucknall T (2012). Bridging the know-do gap in health care through integrated knowledge translation. Worldviews Evid-Based Nurs.

[29] Wehrens R (2014). Beyond two communities -from research utilization and knowledge translation to co-production?. Public Health.

[30] Green LW, Mercer SL (2001). Can public health researchers and agencies reconcile the push from funding bodies and the pull from communities?. Am J Public Health.

[31] Canadian Institutes of Health Research (CIHR) (2015). A guide to knowledge translation at CIHR: integrated and end of grant approaches.

[32] Fisk NM, Wesselingh SL, Beilby JJ, Glasgow NJ, Puddey IB, Robinson BG, Angus JA, Smith PJ (2011). Academic health science centres in Australia: let’s get competitive. Med J Aust.

[33] National Institute for Health Research. About the Collaborations for Leadership in Applied Health Research and Care (CLAHRCs); 2019. https://clahrcprojects.co.uk/about. Accessed 9 Feb 2019.

[34] Jansen MW, van Oers HA, Middelweerd MD, van de Goor IA, Ruwaard D. Conditions for sustainability of Academic Collaborative Centres for Public Health in the Netherlands: a mixed methods design. Health Res Policy Sys. 2015;13:36. 10.1186/s12961-015-0026-7. PMC4546140 26293332

[35] Frank L, Forsythe L, Ellis L, Schrandt S, Sheridan S, Gerson J, Konopka K, Daugherty S (2015). Conceptual and practical foundations of patient engagement in research at the Patient-Centered Outcomes Research Institute. Qual Life Res.

[36] Lomas J (2000). Using linkage and exchange to move research into policy at a Canadian Foundation. Health Aff.

[37] Grant MJ, Booth A (2009). A typology of reviews: an analysis of 14 review types and associated methodologies. Health Inf Libr J.

[38] Domecq JP, Prutsky G, Elraiyah T, Wang Z, Nabhan M, Shippee N, Brito JP, Boehmer K, Hasan R, Firwana B, Erwin P (2014). Patient engagement in research: a systematic review. BMC Health Serv Res.

[39] Shippee ND, Domecq Garces JP, Prutsky Lopez GJ, Wang Z, Elraiyah TA, Nabhan M, Brito JP, Boehmer K, Hasan R, Firwana B, Erwin PJ (2015). Patient and service user engagement in research: a systematic review and synthesized framework. Health Expect.

[40] Patton M (2002). Qualitative research and evaluation methods.

[41] Tong A, Sainsbury P, Craig J (2007). Consolidated criteria for reporting qualitative research (COREQ): a 32-item checklist for interviews and focus groups. Int J Qual Health Care.

[42] Salganik MJ, Heckathorn DD (2004). Sampling and estimation in hidden populations using respondent-driven sampling. Sociol Methodol.

[43] Braun V, Clarke V (2006). Using thematic analysis in psychology. Qual Res Psychol.

[44] Bowen S, Higginbottom G, Liamputtong P (2015). The relationship between engaged scholarship, knowledge translation and participatory research. Participatory qualitative research methodologies in health.

[45] Van de Ven AH (2007). Engaged scholarship: a guide for organizational and social research.

[46] Voorberg WH, Bekkers VJ, Tummers LG (2015). A systematic review of co-creation and co-production: embarking on the social innovation journey. Public Manag Rev.

[47] Piggot-Irvine E, Zornes D (2016). Developing a framework for research evaluation in complex contexts such as action research. SAGE Open.

[48] Chevalier JM, Buckles DJ (2013). Chapter 1: action research history, in participatory action research: theory and methods for engaged inquiry.

[49] Thomas A, Menon A, Boruff J, Rodriguez AM, Ahmed S (2014). Applications of social constructivist learning theories in knowledge translation for healthcare professionals: a scoping review. Implement Sci.

[50] Bechara JP, Van de Ven AH, Van de Ven AH (2007). Chapter 2: philosophy of science underlying engaged scholarship. Engaged scholarship: a guide for organizational and social research.

[51] Nowotny H, Scott P, Gibbons M (2003). Mode 2 revisited: the new production of knowledge. Minerva.

[52] Kuutti K (2007). Design research, disciplines, and new production of knowledge.

[53] Kincheloe JL (2001). Describing the bricolage: conceptualizing a new rigor in qualitative research. Qual Inq.

[54] Latour B (1993). We have never been modern.

[55] Coghlan D (2016). Retrieving a philosophy of practical knowing for action research. Int J Action Res.

[56] Adelman C (1993). Kurt Lewin and the origins of action research. Educ Action Res.

[57] Rimmer TC, Johnson LL (1998). Planned change theories for nursing.

[58] Nguyen T, Graham ID. What’s in a name? Exploring the differences and similarities among knowledge translation approaches. Knowledge Utilization Colloquium November 8-10, 2017 (Oral Presentation). Melbourne, Australia.

[59] Lomas J (1997). Improving research dissemination and uptake in the health sector: beyond the sound of one hand clapping.

[60] Graham ID, Tetroe J, The KT Theories Research Group (2007). Some theoretical underpinnings of knowledge translation. Acad Emerg Med.

[61] Graham ID (2007). Knowledge translation at CIHR: under new administration.

[62] Graham ID, Bernstein A (2007). Health research: an excuse not to act as a catalyst for change. Hill Times.

[63] Tetroe J (2007). Knowledge translation at the Canadian Institutes of Health Research: a primer. Focus Tech Brief.

[64] Salsberg J, Macaulay AC, Parry D, Graham ID, Tetroe JM, Pearson A (2014). Chapter 2: guide to integrated knowledge translation research: researcher and knowledge-user collaboration in health research. Turning knowledge into action: practical guidance on how to do integrated knowledge translation research.

[65] Kerner J (2009). Knowledge translation in cancer surveillance: what do we know, how should we share it, and who cares?.

[66] Graham ID, Kothari A, McCutcheon C, Angus D, Lukaris-Banner D, Botting I, Bucknall T, Dunn S, Gagnon M, Gifford W, Godfrey C, Holmes B, Horsley T, Hutchinson A, Jull J, Law S, McLean R, Mrklas K, Nguyen T, Plamodon K, Rycroft-Malone J, Sibald S, Stacey D, Wright D, Yeung E, for the Integrated Translation Research Network (2018). Moving knowledge into action for more effective practice, programmes and policy: protocol for a research programme on integrated knowledge translation. Implement Sci.

[67] Kothari A, Wathen NC (2013). A critical second look at integrated knowledge translation. Health Policy.

[68] Gagliardi AR, Kothari A, Graham ID (2016). Research agenda for integrated knowledge translation (IKT) in healthcare: what we know and do not yet know. J Epidemiol Community Health.

[69] Flagg JL, Lane JP, Lockett MM (2013). Need to Knowledge (NtK) Model: an evidence based framework for generating technological innovations wtih socio-economic impacts. Implement Sci.

[70] Lane JP, Flagg JL (2010). Traslating three states of knowledge-discovery, invention and innovation. Implement Sci.

[71] Holmes C, McDonald F, Jones M, Graham J (2016). Knowledge translation: moving proteomics science to innovation in society. OMICS.

[72] Barber T, Sharif B, Teare S, Miller J, Shewchuk B, Green LA, Marlett N, Cibere J, Mrklas K, Wasylak T, Li LC, Campbell-Scherer D, Marshall DA (2019). A qualitative study to elicit patients’ and primary care physicians’ perspectives on the use of a self-management mobile health application for knee osteoarthritis. BMJ Open.

[73] The Service and Market Oriented Transport Research Group (SAMOT). Developing public transport through multidisciplinary and benefit-oriented research – Final Report VINN Excellence Center Stage 1. https://www5.kau.se/sites/default/files/Dokument/subpage/2011/08/samot_stage_1_pdf_10169.pdf. Accessed 6 Feb 2019.

[74] Boyer EL (1990). Scholarship reconsidered: the priorities of the professoriate. special report.

[75] Bowen S, Graham ID (2013). From knowledge translation to ‘engaged scholarship:’ promoting research relevance and utilization. Arch Phys Med Rehabil.

[76] Campbell HJ, Vanderhoven D (2016). Knowledge that matters: realising the potential of co-production.

[77] Kellogg Commission on the Future of State and Land-Grant Universities (2001). Returning to our Roots: Executive summaries of the reports of the Kellogg Commission on the future of state and land grant universities.

[78] Anderson JA (2014). Engaged learning, engaged scholarship: a struggle for the soul of higher education. Northwest J Comm.

[79] Harkavy I (2006). The role of universities in advancing citizenship and social justice in the 21st century. Educ Citizenship Soc Justice.

[80] Hartley M, Saltmarsh J, Clayton P (2010). Is the civic engagement movement changing higher education?. Br J Educ Stud.

[81] Bowen S (2014). A school of epidemiology and public health for the 21st Century: the role of engaged scholarship.

[82] Cox D, Fitzgerald HE, Burack C, Siefer S (2010). History of the scholarship of engagement movement. Handbook on engaged scholarship: contemporary landscapes, future directions.

[83] Glass CR, Fitzgerald HE, Fitzgerald HE, Burack C, Siefer S (2010). Engaged scholarship: historical roots, contemporary challenges. Handbook on engaged scholarship: contemporary landscapes, future directions.

[84] Stanton TE (2008). New times demand new scholarship. Educ Citizenship Soc Justice.

[85] Maxwell N (2014). How universities can help to create a wiser world: the urgent need for an academic revolution.

[86] MacIntyre A (1990). Three rival versions of moral enquiry: encyclopedia, genealogy, and tradition.

[87] Van de Ven AH, Zlotkowski E (2005). Toward a scholarship of engagement: a dialogue between Andy Van de Ven and Edward Zlotkowski. Acad Manag Learn Educ.

[88] Gibbons M (1999). Science’s new social contract with society. Nature.

[89] Hessels L, van Lente H (2008). Re-thinking new knowledge production: a literature review and research agenda. Res Policy.

[90] Nowotny H, Peter S, Michael G, Scott P (2001). Re-thinking science: knowledge and the public in an age of uncertainty.

[91] Carayannis EG, Campbell DFJ, Rehman SS (2016). Mode 3 knowledge production: systems and systems theory, clusters and networks. J Innov Entrep.

[92] Schoonmaker MG, Carayannis EG (2013). Mode 3: a proposed classification scheme for the knowledge economy and society. J Knowl Econ.

[93] Ostrom E, Baugh WH (1973). Community organization and the provision of police services.

[94] Ostrom E, Parks RB, Whitaker GP, Percy SL (1978). The public service production process: a framework for analyzing police services. Policy Stud J.

[95] Parks RB, Baker PC, Kiser L, Oakerson R, Ostrom E, Ostrom V, Percy SL, Vandivort MB, Whitaker GP, Wilson R (1981). Consumers as coproducers of public services: some economic and institutional considerations. Policy Stud J.

[96] Powell K, Kitson A, Hoon E, Newbury J, Wilson A, Beilby J (2013). A study protocol for applying the co-creating knowledge translation framework to a population health study. Implement Sci.

[97] Ramirez R (1999). Value co-production: intellectual origins and implications for practice and research. Strat Manage J.

[98] Rycroft-Malone J, Burton CR, Bucknall T, Graham ID, Hutchinson AM, Stacey D (2016). Collaboration and co-production of knowledge in healthcare: opportunities and challenges. Int J Health Policy Manag.

[99] Filipe A, Renedo A, Marston C (2017). The co-production of what? Knowledge values, and social relations in health care. PLoS Biol.

[100] Cahn E (2001). No more throwaway people: the co-production imperative.

[101] Jasanoff S (2004). States of knowledge: the co-production of science and social order.

[102] Jasanoff S (2004). Science and citizenship: a new synergy. Sci Public Policy.

[103] Realpe A, Wallace LM (2010). What is co-production.

[104] Lewin K (1946). Action research and minority problems. J Soc Issues.

[105] Lewin K. In: Lewin GW, editor. Resolving social conflicts; selected papers on group dynamics. New York: Harper & Row, 1948.

[106] Freire P (1970). The pedagogy of the oppressed.

[107] Tuhiwai Smith L (2012). Decolonizing methodologies: research and Indigenous peoples.

[108] Boaventura de Sousa Santos (2016). Epistemologies of the South: justice against epistemicide.

[109] Hall B, Tandon R, Tremblay C (2015). Strengthening community-university research partnerships: global perspectives.

[110] Muhammad M, Wallerstein N, Sussman AL, Avila M, Belone L, Duran B (2015). Reflections on researcher identity and power: the impact of positionality on community-based participatory research (CBPR) processes and outcomes. Crit Sociol.

[111] Allen ML, Salsberg J, Knot M, LeMaster JW, Felzien M, Westfall JM, Herbert CP, Vickery K, Culhane-Pera KA, Ramsden VR, Zittleman L, Martin RE, Macaulay AC (2017). Engaging with communities, engaging with patients: amendment to the NAPCRG 1998 policy statement on responsible research with communities. Fam Pract.

[112] Argyris C, Schön DA (1978). Organizational learning: a theory of action perspective.

[113] Arnstein SR (1969). A ladder of citizen participation. J Am Inst Plann.

[114] Fals-Borda O, Rahman MA (1991). Action and knowledge: breaking the monopoly with participatory action- research.

[115] Dewey J (1954). The public and its problems.

[116] Dewey J (1957). Reconstruction in philosophy.

[117] Dewey J, Morris D, Shapiro I, Dewey J (1993). The need for a recovery of philosophy. The Political Writings.

[118] Duran E, Duran B (1995). Native American postcolonial psychology.

[119] Green LW (2008). Making research relevant: if it is an evidence-based practice, where’s the practice-based evidence?. Fam Pract.

[120] Habermas J (1971). Knowledge and Human Interest.

[121] Hall B, Reason P, Rowan J (1981). The democratization of research in adult and non-formal education. Human inquiry: a source of new paradigm research.

[122] Hall B, Park P, Brydon-Miller M, Hall B, Jackson T (1993). Introduction. Voices of change: participatory research in the United States and Canada.

[123] Israel BA, Schulz AJ, Parker EA, Becker AB (1998). Review of community-based research: assessing partnership approaches to improve public health. Annu Rev Public Health.

[124] Israel BA, Eng E, Schulz AJ, Parker EA (2005). Methods in community-based participatory research for health.

[125] Israel BA, Eng E, Schulz AJ, Parker EA (2012). Methods in community-based participatory research for health.

[126] Jagosh J, Macaulay AC, Pluye P, Salsberg J, Bush PL, Henderson J, Sirett E, Wong G, Cargo M, Herbert CP, Seifer SD, Green LW, Greenhalgh T (2012). Uncovering the benefits of participatory research: implications of a realist review for health research and practice. Millbank Q.

[127] Jagosh J, Bush PL, Salsberg J, Macaulay AC, Greenhalgh T, Wong G, Cargo M, Green LW, Herbert CP, Pluye P (2015). A realist evaluation of community-based participatory research: partnership synergy, trust building and related ripple effects. BMC Public Health.

[128] Kemmis S, McTaggart R, Nixon R (2013). The action research planner: doing critical participatory action research.

[129] Kuhn TS (1962). The structure of scientific revolutions.

[130] Kuhn TS (1970). The structure of scientific revolutions.

[131] Macaulay AC, Commanda LE, Freeman WL, Gibson N, McCabe ML, Robbins CM, Twohig PL (1999). Participatory research maximises community and lay involvement. BMJ..

[132] Macaulay AC (2017). Participatory research: what is the history? Has the purpose changed?. Fam Pract.

[133] Mercer SL, Green LW, Cargo M, Potter MA, Daniel M, Olds S, Reed-Gross E, Minkler M, Wallerstein N (2008). Reliability-tested guidelines for assessing participatory research projects. Community based participatory research for health: from process to outcomes.

[134] Minkler M, Garcia A, Rubin V, Wallerstein N. Community-Based Participatory Research: A Strategy for Building Healthy Communities and Promoting Health through Policy Change. 2012. http://www.policylink.org/sites/default/files/CBPR.pdf. Accessed 26 Feb 2020.

[135] Minkler M, Salvatore A. 10 Participatory Approaches for Study Design and Analysis in Dissemination and Implementation Research. In: Ross C. Brownson, Graham A. Colditz, and Enola K. Proctor, editors. Dissemination and Implementation Research in Health: Translating Science to Practice. Oxford University Press Online; 2012.

[136] Minkler M, Wallerstein N (2008). Community-based participatory research for health: from processes to outcomes.

[137] Minkler M (2005). Community-based research partnerships: challenges and opportunities. J Urban Health.

[138] Minkler M, Vasquez VB, Warner JR, Steussey H, Facente S (2006). Sowing the seeds for sustainable change: a community-based participatory research partnership for health promotion in Indiana, USA and its aftermath. Health Promot Int.

[139] Minkler M, Freudenberg N, Fitzgerald HS, Burack C, Siefer S (2010). From community-based participatory research to policy change. Handbook on engaged scholarship: contemporary landscapes, future directions.

[140] Ramsden V, Salsberg J, Herbert C, Westfall J, LeMaster J, Macaulay AC (2017). Patient and community oriented research: how is authentic engagement identified in grant applications?. Can Fam Phys.

[141] Salsberg J, Parry D, Pluye P, Macridis S, Herbert CP, Macaulay AC (2015). Successful strategies to engage research partners for translating evidence into action in community health: a critical review. J Environ Public Health.

[142] Salsberg J, Macridis S, Garcia Bengoechea E, Macaulay AC, Moore S (2017). The shifting dynamics of social roles and project ownership over the lifecycle of a community-based participatory research project. Fam Pract.

[143] Salsberg J, Merati N (2018). Participatory health research in North America: from community engagement to evidence-informed practice, in participatory health research: voices from around the world.

[144] Selener D (1997). Participatory action research and social change.

[145] Viswanathan M, Ammerman A, Eng E, Garlehner G, Lohr KN, Griffith D, Rhodes S, Samuel-Hodge C, Maty S, Lux L, Webb L (2004). Community-based participatory research: assessing the evidence. Evid Rep Technol Assess.

[146] Wallerstein NB, Duran B, Minkler M, Wallerstein N (2003). The conceptual, historical and practice roots of community based participatory research and related participatory traditions. Community-based participatory research for health.

[147] Wallerstein NB, Duran B (2006). Using community-based participatory research to address health disparities. Health Promot Pract.

[148] Wallerstein NB, Duran B (2010). Community-based participatory research contributions to intervention research: the intersection of science and practice to improve health equity. Am J Public Health.

[149] Wallerstein NB, Duran B, Oetzel J, Minkler M (2018). Community-based participatory research for health: advancing social and health equity.

[150] Wright MT, Cook T, Springett J, Roche B, Stern T, Townsend A, Rauch F, Schuster A (2013). The international collaboration for participatory health research: legitimating the science and ensuring quality. Action research, innovation and change: International perspectives across disciplines.

[151] Foote Whyte W (1991). Participatory action research.

[152] Chambers R (1992). Rural apprasial: rapid, relaxed and participatory.

[153] Cornwall A, Jewkes R (1995). What is participatory research?. Soc Sci Med.

[154] Hakala J, Ylijoki OH (2001). Research for whom? Research orientations in three academic cultures. Organization.

[155] Crotty M, Crotty M (1998). Introduction: the research process. The foundations of social research: meaning and perspective in the research process.

[156] Oetzel JG, Wallerstein N, Duran B, Sanchez-Youngman S, Nguyen T, Woo K, Wang J, Schulz A, Kaholokula KA, Israel B, Alegria M (2018). Impact of participatory health research: a test of the community-based participatory research conceptual model. Biomed Res Int.

[157] Canadian Academy of Health Sciences (2018). Academic recognition of team science: how to optimize the canadian academic system.

[158] Boivin A, L’Espérance A, Gauvin F-P, Dumez V, Macaulay AC, Lehoux P, Abelson J (2018). Patient and public engagement in research and health system decision-making: a systematic review of evaluation tools. Health Expect.

[159] Greenhalgh T, Fahy N (2015). Research impact in the community-based health sciences: an analysis of 162 case studies from the 2014 UK Research Excellence Framework. BMC Med.

[160] Camden C, Shikako-Thomas K, Nguyen T, Graham E, Thomas A, Sprung J, Morris C, Russell DJ (2015). Engaging stakeholders in rehabilitation research: a scoping review of strategies used in partnerships and evaluation of impacts. Disabil Rehabil.

