# Supplementary material for: How does integrated knowledge translation (IKT) compare to other collaborative research approaches to generating and translating knowledge? Learning from experts in the field
Source: Health Res Policy Syst. 2020 Mar 30;18:35. doi: 10.1186/s12961-020-0539-6 (PMC7106699; doi:10.1186/s12961-020-0539-6)
Supplement: Supplementary file 1 — Additional file 1. Interview guide. [file 12961_2020_539_MOESM1_ESM.docx]

***Additional file 1: Interview guide***

1. What KT approach (modes of knowledge production (Mode 1, 2, &3), engaged scholarship, participatory action research, community based research, and co-production) would you like to discuss in comparison with IKT?
2. What are the differences and similarities in theoretical underpinnings, values, and principles of your chosen approach or approaches in comparison to IKT?
3. What are the defining characteristics (i.e. what is distinct, different, or unique) about your chosen approach or approaches in comparison to IKT?
4. How do you define partnership and engagement (i.e. what is the role of researchers, partners, stakeholders, knowledge users etc.)?
5. What are the historical roots of your chosen approach or approaches?
6. What are the germinal texts of your chosen approach or approaches?
7. Who are the key experts in your chosen approach or approaches?
8. What is your availability within the next two weeks for a phone interview to discuss your responses?
