# Supplementary material for: How does integrated knowledge translation (IKT) compare to other collaborative research approaches to generating and translating knowledge? Learning from experts in the field
Source: Health Res Policy Syst. 2020 Mar 30;18:35. doi: 10.1186/s12961-020-0539-6 (PMC7106699; doi:10.1186/s12961-020-0539-6)
Supplement: Supplementary file 2 — Additional file 2. Key literature. [file 12961_2020_539_MOESM2_ESM.docx]

***Additional file 2: Key literature***

| **Integrated Knowledge Translation** | Bowen S, Graham ID. Integrated knowledge translation. In: Straus SE, Tetroe J, Graham ID, editors. Knowledge translation in health care: moving evidence to practice. West Sussex, UK: John Wiley & Sons; 2013. p. 14–23.  Canadian Institutes of Health Research (CIHR). A guide to knowledge translation at CIHR: integrated and end of grant approaches. http://www.cihr-irsc.gc.ca/e/45321.html (2015). Accessed 9 February 2018.  Denis JL, Lomas J. Convergent evolution: The academic and policy roots of collaborative research. J Health Serv Res Policy*,* 2003; 8 Suppl 2:1-6.  Gagliardi AR, Berta W, Kothari A, Boyko J, Urquhart R. Integrated knowledge translation (IKT) in health care: a scoping review. Implement Sci. 2015; 71(2):105-106. doi.10.1186/s13012-016-0399-1.  Gagliardi AR, Kothari A, Graham ID. Research agenda for integrated knowledge translation (IKT) in healthcare: what we know and do not yet know. J Epidemiol Community Health. 2016; 71(2):105-106.  Graham ID, Bernstein A. Health research: An excuse not to act as a catalyst for change. Hill Times. 2007;30:1.  Graham ID, Logan J, Harrison MB, Straus SE, Tetroe J, Caswell W, Robinson N. Lost in knowledge translation: time for a map? J Contin Educ Health Prof. 2006;26:13–24  Graham ID, Tetroe J. CIHR research: How to translate health research knowledge into effective healthcare action. Healthc Q. 2007;10(3):20-22.  Graham ID, Tetroe J, Pearson A. Turning knowledge into action: practical guidance on how to do integrated knowledge translation research. 1^st^ ed. Philadelphia, PA: Lippincott Williams & Wilkins; 2014.  Graham ID, Kothari A, McCutcheon C, and the Integrated Knowledge Translation Research Network Project Leads. Moving knowledge into action for more effective practice, programmes and policy: protocol for a research programme on integrated knowledge translation. Implement Sci. 2018; 13(22). doi: [10.1186/s13012-017-0700-y](https://doi-org.proxy.bib.uottawa.ca/10.1186/s13012-017-0700-y).  Kothari A, McCutcheon C, Graham ID, for the IKT Research Network. Defining integrated knowledge translation and moving forward: A response to recent commentaries. Int J Health Policy Manag. 2017;6:1-2.  Kothari A, Wathen NC. A critical second look at integrated knowledge translation. Health Policy. 2013;109(2):187–91.  Lomas J. Improving research dissemination and uptake in the health sector: beyond the sound of one hand clapping. McMaster Univeristy Centre for Health Economics and Policy Analysis: Hamilton, Ontario; 1997, p.1-45.  Lomas J. Using linkage and exchange to move research into policy at a Canadian Foundation. Health Aff. 2000; 19:236–240.  Lomas J. Using research to inform healthcare managers’ and policy makers’ questions: from summative to interpretive synthesis. Healthcare Policy. 2005;1(1):55.  Straus SE, Tetroe J, Graham ID. Knowledge translation in healthcare: Moving from evidence to practice. 2nd ed. West Sussex, UK: John Wiley & Sons; 2013. |
| --- | --- |
| **Engaged Scholarship** | Anderson JA. Engaged learning, engaged scholarship: A struggle for the soul of higher education. Northwest J Comm. 2014;42(1):143-66.  Boyer EL. Scholarship reconsidered: The priorities of the professoriate. Special report. Princeton, N.J.: Carnegie Foundation for the Advancement of Teaching; 1990.  Boyer EL. The scholarship of engagement. Journal of Public Service and Outreach. 1996; 1(1):11–21.  Bowen S, Graham ID. From knowledge translation to ‘engaged scholarship:’ promoting research relevance and utilization. Arch Phys Med Rehabil. 2013;94 Suppl 1:S3–S8.  Bowen, S. The relationship between engaged scholarship, knowledge translation and participatory research. In: Higginbottom G, Liamputtong P, editors. Participatory qualitative research methodologies in health. Sage Publications: Los Angeles, CA; 2015. p.183-199.  Bowen, S. A School of Epidemiology and Public Health for the 21st Century: The Role of Engaged Scholarship. Ottawa: Department of Epidemiology and Community Medicine University of Ottawa; 2014, p.1-38.  Cox D. History of the Scholarship of Engagement Movement. In: Fitzgerald HE, Burack C, Siefer S, editors. Handbook on Engaged Scholarship: Contemporary landscapes, future directions. East Lansing, MI: Michigan State University Press; 2010.  Glass CR, Fitzgerald HE. Engaged Scholarship: Historical roots, contemporary challenges. In: Fitzgerald HE, Burack C, Siefer S, editors. Handbook on Engaged Scholarship: Contemporary landscapes, future directions. East Lansing, MI: Michigan State University Press; 2010.  Kellogg Commission on the Future of State and Land-Grant Universities. Returning to our Roots: Executive summaries of the reports of the Kellogg Commission on the future of state and land grant universities. <http://www.aplu.org> (2001). Accessed 9 February, 2018.  Stanton TE. New times demand new scholarship. Educ Citizsh Soc Justice. 2008; 3:19  Van de Ven AH, Johnson P. Knowledge for theory and practice. Acad Manag Rev. 2006; 31 (4) 802–821.  Van de Ven AH. Engaged scholarship: A guide for organizational and social research. Oxford, UK: Oxford University Press; 2007.  Van de Ven AH, Zlotkowski E. (2005). Toward a scholarship of engagement: A dialogue between Andy Van de Ven and Edward Zlotkowski. Acad Manag Learn Educ. 2005;4:355-62. |
| **Mode 2 Research** | Carayannis EG, Campbell DFJ, Rehman SS. Mode 3 knowledge production: systems and systems theory, clusters and networks. Journal of Innovation and Entrepreneurship 2016;5(1):17.  Carayannis EG, Barth TD, Campbell DF. The quintuple helix innovation model: Global warming as a challenge and driver for innovation. Journal of Innovation and Entrepreneurship. 2012;1(1):2.  Estabrooks CA, Norton P, Birdsell JM, Newton MS, Adewale AJ, Thornley R. Knowledge translation and research careers: Mode I and Mode II activity among health researchers. Res Policy. 2008;37(6-7):1066-78.  Gibbons M, Limoges C, Nowotny H, Schwartzman S, Scott P, Trow M. The new production of knowledge: The dynamics of science and research in contemporary societies. London: SAGE Publications; 1994.  Nowotny, H, Peter S, Michael G, Scott P. Re-thinking science: Knowledge and the public in an age of uncertainty. Cambridge: Polity, 2001.  Nowotny H, Scott P, Gibbons M. 2003. Mode 2 revisited: the new production of knowledge. Minerva. 2003;41:179–94.  Schoonmaker, MG, Carayannis, EG. Mode 3: A proposed classification scheme for the knowledge economy and society. Journal of the Knowledge Economy. 2013;4(4), 556-77. |
| **Co-production** | Cahn E. No More Throwaway People: The Co-production Imperative. Washington DC: Essential Books; 2001.  Campbell HJ, Vanderhoven D. Knowledge that matters: realising the potential of co-production. http://eprints.whiterose.ac.uk/99657/1/Final%20Report%20-%20Co-Production%20-%20%202016-01-20.pdf (2016). Accessed 9 February 2018.  Bovaird T. Beyond engagement and participation: User and community coproduction of public services. Public Adm Rev. 2007;67(5):846-60.  Boyle D, Harris M. The challenge of co-production–How equal partnership between professionals and the public are crucial to improving public services. London, England: NESTA; 2009.  Brandsen T, Pestoff V. Co-production, the third sector and the delivery of public services: An introduction. Public Manag Rev. 2006;8(4):493-501.  Filipe A, Renedo A, Marston C. The co-production of what? Knowledge values, and social relations in health care. PLoS Biol. 2017;15(5):e2001403.  Jasanoff, S. States of knowledge: The co-production of science and social order. 1st ed. London: Routledge; 2004.  Jasanoff, S. Science and citizenship: a new synergy. Science and Public Policy. 2004;31(2):90–94.  Lavis JN, Lomas J, Hamid M, Nelson KS. Assessing country-level efforts to link research to action. Bulletin of the World Health Organization. 2006;84:620-8.  Ostrom E, Baugh WH. Community organization and the provision of police services. Beverly Hills: Sage Publications; 1973.  Ostrom E, Parks RB, Whitaker GP, Percy SL. The public service production process: a framework for analyzing police services. Policy Stud J. 1978;7 Suppl 1:381-9.  Ostrom E. Crossing the great divide: coproduction, synergy, and development. World Dev. 1996;24(6):1073-87.  Parks RB, Baker PC, Kiser L, Oakerson R, Ostrom E, Ostrom V, Percy SL, Vandivort MB, Whitaker GP, Wilson R. Consumers as coproducers of public services: Some economic and institutional considerations. Policy Stud J. 1981;9(7):1001-11.  Powell K, Kitson A, Hoon E, Newbury J, Wilson A, Beilby J. A study protocol for applying the co-creating knowledge translation framework to a population health study. Implement Sci. 2013;8:98.  Ramaswamy V, Gouillart F. Building the co-creative enterprise. Harv Bus Rev. 2010;88.  Ramirez R. Value co-production: intellectual origins and implications for practice and research. Strat Mgmt J. 1999; 20: 49-65.  Realpe A, Wallace LM. What is co-production. London: The Health Foundation. 2010:1-1.  Rycroft-Malone J, Burton CR, Bucknall T, Graham ID, Hutchinson AM, Stacey D. Collaboration and co-production of knowledge in healthcare: opportunities and challenges. [Int J Health Policy Manag](https://www.ncbi.nlm.nih.gov/labs/journals/int-j-health-policy-manag/new/2017-09-28/). 2016;5(4):221.  Von Hippel E. Cooperation between rivals: informal know-how trading. Res Policy. 1987;16(6):291-302.  Voorberg WH, Bekkers VJ, Tummers LG. A systematic review of co-creation and co-production: Embarking on the social innovation journey. Public Management Review. 2015;17(9):1333-57.  Wehrens R. Beyond two communities -from research utilization and knowledge translation to co-production? Public Health. 2014;128(6):545 - 51. |
| **Participatory Research** | Allen ML, Salsberg J, Knot M, LeMaster JW, Felzien M, Westfall JM, Herbert CP, Vickery K, Culhane-Pera KA, Ramsden VR, Zittleman L, Martin RE, Macaulay AC. Engaging with communities, engaging with patients: amendment to the NAPCRG 1998 Policy Statement on Responsible Research With Communities. Fam Pract. 2017; 34(3):313-21.  Arnstein SR. A ladder of citizen participation. J Am Inst Plann.1969;35(4):216-24.  Argyris C, Schön DA. Organizational learning: A theory of action perspective. Reading, MA: Addison-Wesley Publishing Company; 1978.  Barber B. Strong democracy: Participatory politics for a new age. Berkeley, CA.: University of California Press; 1984.  Cargo M, Mercer SL. The value and challenges of participatory research: strengthening its practice. Annu Rev Public Health 2008; 29: 325–50.  Chambers R. Rural development: Putting the last first. Edinburgh Gate, England: Addison Wesley Longman Ltd; 1983.  Dewey J. The public and Its problems. 2nd ed. Athens: Swallow Press / Ohio University Press; 1954/1927.  Dewey J. Reconstruction in philosophy. Enlarged ed. Boston, MA: Beacon Press;1957.  Dewey J. The Need for a Recovery of Philosophy. In: Morris D, Shapiro I, Dewey J, editors. The Political Writings. Indianapolis, IN: Hackett Publishing Company; 1993.  Fals-Borda O, Rahman MA. Action and Knowledge: Breaking the Monopoly with Participatory Action- Research. New York: Apex Press; London: Intermediate Technology Publications; 1991.  Freire P. The Pedagogy of the Oppressed. New York, NY: Herder and Herder; 1970.  Green LW. Making research relevant: if it is an evidence-based practice, where’s the practice-based evidence? Fam Pract. 2008; 25: i20–i24  Green LW, Mercer SL. Can public health researchers and agencies reconcile the push from funding bodies and the pull from communities? Am J Public Health. 2001; 91:1926–29.  Green LW, George A, Daniel M, Frankish J, Herbert CJ, et al. 1995. Participatory Research in Health Promotion. Ottawa: R. Soc. Can.  Greenhalgh T, Jackson C, Shaw S, Janamian T. Achieving research impact through co-creation in community-based health services: literature review and case study. Milbank Q. 2016;94(2):392–429.  Greenhalgh T, Fahy N. Research impact in the community-based health sciences: an analysis of 162 case studies from the 2014 UK Research Excellence Framework. BMC Medicine. 2015;13(1):232.  Greenwood DJ, Levin M. Introduction to action research: Social research for social change. Thousand Oaks, CA: SAGE Publications; 1998.  Habermas J. Knowledge and Human Interest. Boston, MA: Beacon Press; 1971.  Hall B. (1981). The democratization of research in adult and non-formal education. In: Reason P, Rowan J, editors. Human inquiry: A source of new paradigm research. New York: John Wiley & Sons; 1981. p.447-456.  Hall B. Introduction. In: Park P, Brydon-Miller M, Hall B, Jackson T, editors. Voices of change: Participatory research in the United States and Canada. Toronto: OISE Press; 1993. p. xiii–xxii.  Heron J, Reason P. The practice of cooperative inquiry: research “with” rather than “on” people. In Handbook of Action Research: Participative Inquiry and Practice, ed. P Reason, H Bradbury, pp. 179–88. Thousand Oaks, CA: Sage; 2001.  Israel BA, Schulz AJ, Parker EA, Becker AB. Review of community-based research: Assessing partnership approaches to improve public health. Annu Rev Public Health. 1998;19:173–202.  Israel BA, Eng E, Schulz AJ, Parker EA. Methods in community-based participatory research for health. San Francisco, CA: John Wiley & Sons; 2005.  Israel BA, Eng E, Schulz AJ, Parker EA. Methods in community-based participatory research for health. 2nd ed. San Francisco, CA: John Wiley & Sons; 2012.  Jagosh J, MacAulay AC, Pluye P, Salsberg J, Bush PL, Henderson J, Sirett E, Wong G, Cargo M, Herbert CP, Seifer SD, Green LW, Greenhalgh T. Uncovering the benefits of participatory research: Implications of a realist review for health research and practice. Millbank Q. 2012; 90(2): 311-46  Jagosh J, Bush PL, Salsberg J, Macaulay AC, Greenhalgh T, Wong G, Cargo M, Green LW, Herbert CP, Pluye P. A realist evaluation of community-based participatory research: partnership synergy, trust building and related ripple effects. BMC Public Health. 2015;15(1):725.  Jones L, Wells K. Strategies for Academic and Clinician Engagement in Community-Participatory Partnered Research. JAMA. 2007; 297(4): 407-410  Kemmis S, McTaggart R, Nixon R. The action research planner: Doing critical participatory action research. Springer Science & Business Media; 2013.  Kuhn TS. The structure of scientific revolutions. 1st ed. Chicago: University of Chicago Press; 1962.  Labonte R, Feather J. Handbook on using stories in health promotion practice. Ottawa, ON: Health Canada. 1996*.*  Lincoln YS. Sympathetic connections between qualitative methods and health research*.* Qual Health Res. 1992;2(4):375-91.  Lewin K. Action research and minority problems. J Soc Issues. 1946;2(4): 34-46  Lewin K. In: Lewin GW, editor. Resolving social conflicts; Selected papers on group dynamics. New York, NY: Harper & Row, 1948.  Macaulay AC, Commanda LE, Freeman WL, Gibson N, McCabe ML, Robbins CM, Twohig PL. Participatory research maximises community and lay involvement. BMJ. 1999;319(7212):774-8.  Macaulay AC. Participatory research: What is the history? Has the purpose changed?. Fam Pract. 2017;34(3):256-58.  MacDonald C. Understanding participatory action research: A qualitative research methodology option. The Canadian Journal of Action Research. 2012;13(2):34-50.  Maguire P. Doing participatory action research: A feminist approach. Massachusetts: University of Massachusetts Press; 1987.  Maslow, A. H. (1998). Maslow on management. Toronto, ON: John Wiley and Sons; 1998.  McTaggart R. Principles for participatory action research. Adult Educ Q. 1991;41(3): 168-187.  Mercer SL, Green LW, Cargo M, Potter MA, Daniel M, Olds S, & Reed-Gross E. Reliability-tested guidelines for assessing participatory research projects. In Minkler M & Wallerstein N (Eds), Community based participatory research for health: From process to outcomes (pp. 407-418). Hoboken, NJ: Wiley; 2008.  Minkler M, Wallerstein N. Part one: Introduction to community-based participatory research. In: Minkler M, Wallerstein N, editors. Community-based participatory research for health. San Francisco, CA: Jossey-Bass; 2003. p. 5-24.  Minkler M, Wallerstein N. Community-based participatory research for health: From processes to outcomes. 2nd ed. San Francisco, CA: Jossey-Bass; 2008.  Minkler M. Ethical Challenges for the “Outside Researcher” in Community-Based Participatory Research. Health Educ Behav. 2004; 31(6): 684-697  Minkler M. Community-based research partnerships: challenges and opportunities. J Urban Health. 2005; 82:ii3–12.  Minkler M, Vasquez VB, Warner JR, Steussey H, Facente S. 2006. Sowing the seeds for sustainable change: a community-based participatory research partnership for health promotion in Indiana, USA and its aftermath. Health Promot Int. 2006; 21:293–300  Minkler M, Freudenberg N. From Community-based participatory research to policy change. In: Fitzgerald HS, Burack C, Siefer S, editors Handbook on Engaged Scholarship: Contemporary landscapes, future directions. East Lansing, MI: Michigan State University Press; 2010.  Ramsden V, Salsberg J, Herbert C, Westfall J, LeMaster J, Macaulay AC. Patient and community oriented research: How is authentic engagement identified in grant applications?. [Can Fam Physician. 2017;63(1):74-76.](http://www.cfp.ca/content/63/1/74.full)  Reason P. (Ed.). (1994). Participation in Human Inquiry. London: Sage; 1994.  Reason P. The Co-operative Inquiry Group. In P. Reason (ed.) Human Inquiry in Action. Developments in New Paradigm Research. Newbury Park, CA: Sage; 1988.  Reason P, Bradbury H (Eds). The Sage handbook of action research. Participative Inquiry and Practice. 2nd ed. London: Sage; 2008.  Salsberg J, Parry D, Pluye P, Macridis S, Herbert CP, Macaulay AC. Successful strategies to engage research partners for translating evidence into action in community health: a critical review. J Environ Public Health. 2015. doi.org/10.1155/2015/191856  Salsberg J, Macridis S, Garcia Bengoechea E, Macaulay AC, Moore S. The Shifting Dynamics of Social Roles and Project Ownership over the Lifecycle of a Community-Based Participatory Research Project. [Fam Pract. 2017; 34(3): 305-12.](https://academic.oup.com/fampra/article/3038266/The-shifting-dynamics-of-social-roles-and-project)  Salsberg J, Merati N. Participatory Health Research in North America: From Community Engagement to Evidence-Informed Practice, in Participatory Health Research: Voices from around the world (in press), M.T.W.a.K. Kongats, Editor. San Fransisco, CA: Springer, CA; 2018.  Selener D. Participatory Action Research and Social Change. Ithaca, NY: Cornell University; 1997.  Tandon SD, Phillips K, Bordeaux BC, Bone L, Brown PB, Cagney KA, Gary TL, Kim M, Levine DM, Price E, Sydnor KD. A vision for progress in community health partnerships. Prog Community Health Partnersh. 2007;1(1):11-30.  Viswanathan M, Ammerman A, Eng E, Garlehner G, Lohr KN, Griffith D, Rhodes S, Samuel-Hodge C, Maty S, Lux L, Webb L. Community-based participatory research: assessing the evidence. Evid Rep Technol Assess. 2004; 99:1–8.  Viswanathan M, Ammerman A, Eng E, Gartlehner G, Lohr KN, Griffith D, Rhodes S, Samuel-Hodge C, Maty S, Lux, L, Webb L, Sutton SF, Swinson T, Jackman A, Whitener L. (2004). Community-Based Participatory Research: Assessing the Evidence. Evidence Report/Technology Assessment No. 99 (Prepared by RTI–University of North Carolina Evidence-based Practice Center under Contract No. 290-02-0016). AHRQ Publication 04-E022- 2. Rockville, MD: Agency for Healthcare Research and Quality.  Wallerstein NB, Duran B. Using community-based participatory research to address health disparities. Health Promot Pract. 2006;7:312–23  Wallerstein N, Duran B. The conceptual, historical and practice roots of community  based participatory research and related participatory traditions. In: Minkler M, Wallerstein N, editors. Community-based participatory research for health. San Francisco, CA: Jossey-Bass; 2003. p.25-47.  Wallerstein NB, Duran B, Oetzel J, Minkler M. Community-based participatory research for health: Advancing social and health equity. 3rd ed. San Francisco, CA: Jossey-Bass; 2017.  Whyte WF. Participatory action research. Thousand Oaks, CA: SAGE Publications; 1991. |
